# Alterations in the HLA-B*57:01 Immunopeptidome by Flucloxacillin and Immunogenicity of Drug-Haptenated Peptides

**DOI:** 10.3389/fimmu.2020.629399

**Published:** 2021-02-09

**Authors:** Montserrat Puig, Suryatheja Ananthula, Ramesh Venna, Swamy Kumar Polumuri, Elliot Mattson, Lacey M. Walker, Marco Cardone, Mayumi Takahashi, Shan Su, Lisa F. Boyd, Kannan Natarajan, Galina Abdoulaeva, Wells W. Wu, Gregory Roderiquez, William H. Hildebrand, Serge L. Beaucage, Zhihua Li, David H. Margulies, Michael A. Norcross

**Affiliations:** ^1^ Laboratory of Immunology, Office of Biotechnology Products, Center for Drugs Evaluation and Research, Food and Drug Administration, Silver Spring, MD, United States; ^2^ Division of Applied Regulatory Science, Office of Translational Science, Center for Drugs Evaluation and Research, Food and Drug Administration, Silver Spring, MD, United States; ^3^ Laboratory of Biological Chemistry, Office of Biotechnology Products, Center for Drugs Evaluation and Research, Food and Drug Administration, Silver Spring, MD, United States; ^4^ Molecular Biology Section, Laboratory of Immune System Biology, National Institute of Allergy and Infectious Diseases, National Institutes of Health, Bethesda, MD, United States; ^5^ Facility for Biotechnology Resources, Center for Biologics Evaluation and Research, Food and Drug Administration, Silver Spring, MD, United States; ^6^ Department of Microbiology and Immunology, School of Medicine, University of Oklahoma Health Sciences Center, Oklahoma City, OK, United States

**Keywords:** flucloxacillin, HLA-B*57:01, drug hypersensitivity, hapten, immunogenicity, transgenic mice

## Abstract

Neoantigen formation due to the interaction of drug molecules with human leukocyte antigen (HLA)-peptide complexes can lead to severe hypersensitivity reactions. Flucloxacillin (FLX), a β-lactam antibiotic for narrow-spectrum gram-positive bacterial infections, has been associated with severe immune-mediated drug-induced liver injury caused by an influx of T-lymphocytes targeting liver cells potentially recognizing drug-haptenated peptides in the context of HLA-B*57:01. To identify immunopeptidome changes that could lead to drug-driven immunogenicity, we used mass spectrometry to characterize the proteome and immunopeptidome of B-lymphoblastoid cells solely expressing HLA-B*57:01 as MHC-I molecules. Selected drug-conjugated peptides identified in these cells were synthesized and tested for their immunogenicity in HLA-B*57:01-transgenic mice. T cell responses were evaluated *in vitro* by immune assays. The immunopeptidome of FLX-treated cells was more diverse than that of untreated cells, enriched with peptides containing carboxy-terminal tryptophan and FLX-haptenated lysine residues on peptides. Selected FLX-modified peptides with drug on P4 and P6 induced drug-specific CD8^+^ T cells *in vivo*. FLX was also found directly linked to the HLA K146 that could interfere with KIR-3DL or peptide interactions. These studies identify a novel effect of antibiotics to alter anchor residue frequencies in HLA-presented peptides which may impact drug-induced inflammation. Covalent FLX-modified lysines on peptides mapped drug-specific immunogenicity primarily at P4 and P6 suggesting these peptide sites as drivers of off-target adverse reactions mediated by FLX. FLX modifications on HLA-B*57:01-exposed lysines may also impact interactions with KIR or TCR and subsequent NK and T cell function.

## Introduction

Idiosyncratic drug-induced liver injury (DILI) is a rare but potentially fatal adverse drug reaction and a major obstacle in the development of pharmaceuticals. Understanding the biochemistry and immune pathways that mediate severe adverse drug reactions is critical for identifying treatment and prevention strategies to minimize unwanted off-target drug effects. Although liver injury can result from expected toxicities based on the chemical nature of a drug, idiosyncratic reactions may also manifest in susceptible patients. These reactions are complex and many are considered to be immune-mediated based on (i) the presence of mononuclear cells, including effector CD8^+^ T cells, in liver biopsies of patients experiencing DILI (1, 2), and (ii) the reactivity of these patients’ T cells to drug stimulation *in vitro*, often observed to be dependent on the expression of specific HLA Class-I and II alleles (3–5). Genome-wide association studies in patients with FLX-induced DILI found this adverse reaction to be significantly associated with the expression of HLA-B*57:01 (6) and HLA-B*57:03 (7), allelomorphs that differ by only two amino acid residues. T cell reactivity to FLX was demonstrated *in vitro* to be elicited by both soluble drug and drug-pulsed presenting cells, the latter suggested to be mediated by covalently-linked FLX on protein epitopes (4, 5, 8). FLX-stimulated peripheral blood CD8^+^ and CD4^+^ T cells from HLA-B*57:01^+^ DILI patients primarily in an antigen-processing dependent manner (4). However, T cells from HLA-B*57:01^+^ drug-naïve healthy individuals required soluble FLX in the cultures and rarely responded to drug-pulsed targets (5, 8). Of note, T cell clones from the HLA-B*57:01^+^ patients responded to soluble FLX presented by non-B*57:01 HLA alleles (8).

While there is an accepted association between the immune system and drug-mediated liver injury, the mechanisms by which drugs cause immune cell activation are still poorly understood. Proposed molecular mechanisms to explain drug hypersensitivity are based on drug-peptide interactions. T cell responses to non-covalent drug-peptide complexes are known as the pharmaceutical interaction mechanism, while antigen processing-dependent responses are thought to be driven by drug covalently linked to peptides presented by HLA molecules (9–12). In addition, we and others have described a non-covalent mechanism by which abacavir facilitates the loading of novel self-peptides with changes in the consensus C-terminal anchor residues into HLA-B*57:01, inducing autoimmune-like reactions to the drug-altered antigens (13–15). In this report, using immunoproteomics, we show that FLX treatment of antigen presenting cells expressing HLA-B*57:01 effects changes in the immunoproteome by mechanisms different from those of abacavir. Instead, without affecting the cellular proteome, FLX increased the diversity of the peptide repertoire of sampled proteins favoring the presence of tryptophan in the F-pocket anchor motif. We also identified several peptides on the HLA-B*57:01 immunopeptidome pool with FLX-conjugated in lysine residues (FLX-peptides). These were subsequently characterized by mass spectrometry and chemically synthesized for immunogenicity evaluation in an HLA-B*57:01^+^ transgenic mouse. The immunogenic potential of the FLX-peptides was found to depend on both the peptide sequence as well as the position at which FLX was conjugated.

## Methods

### Reagents

Flucloxacillin (FLX) was obtained from Apotex, UK or Sigma-Aldrich, USA. FLX-conjugated lysine residues were prepared according to the method of Scornet et al. (16) (**Figure S1** and **Supplementary Methods**) and used to synthesize FLX-modified peptides by solid-phase Fmoc chemistry.

### B-Lymphoblastoid Cell Lines

The HLA-B*57:01 full-length sequence for membrane bound HLA (mHLA) was transduced using a lentiviral expression system. From here on, HLA-B*57:01-expressing cells are referred as B721-5701. The expression of soluble HLA-B*57:01 (sHLA) in the HLA class I-negative EBV-transformed B-lymphoblastoid 721.221 cell line was described elsewhere (14).

### Proteomics and LC-MS/MS Analysis

Proteomic characterization of B721-5701 cells is presented in **Supplementary Methods**. Cells were grown in the absence or presence of FLX for 5 days in duplicate cultures. Cells were harvested and proteins extracted and trypsinized. After ion exchange fractionation, peptides were analyzed by LC-MS on a Q-Exactive MS with nano-LC. Data was processed with the PEAKS 8.5 software using Uniprot non-redundant human database (https://www.uniprot.org).

### Generation, Isolation, and Identification of HLA-Bound Peptides

B721.5701 cells expressing mHLA were grown without antibiotics in G-Rex 6-Well Plates to high density for 5 days with or without 150 µg/ml FLX as described in **Supplementary Methods**. Cell lysates were immune-affinity purified using anti-HLA class-I antibody W6/32. Acid-eluted peptides were analyzed using nano-LC-MS/MS with a ThermoFisher Ultimate LC and Fusion Orbitrap MS. Soluble HLA from untreated or FLX-treated cultures was collected from continuous cultures as described in **Supplementary Methods**. Peptides were purified from sHLA/peptide complexes by immuno-affinity purification followed by acid elution and filtration. Peptide identity was determined as detailed in **Supplementary Methods**.

### Peptide Docking Modeling

Details of the protocols applied to modeling peptide interactions with drug, HLA and TCR are described in **Supplementary Methods**. Images were generated using PyMOL (The PyMOL Molecular Graphics System, Version 2.0 Schrödinger, LLC).

### Treatment of Mice

HLA-B*57:01 transgenic/H2-K^b^D^b^ knockout (Tg/KO) mice were generated by backcrossing HLA-B*57:01/H2-D^d^ Tg (17) to *H2-K^b^D^b^* KO C57BL/6 (B6.129P2-*H2-Kb^tm1^H2-Db^tm1^* N12) mice (18) (Taconic Biosciences, Hudson, NY). Tg/KO mice were immunized with 100 µg of FLX-haptenated peptide (FLX-peptide) containing immune adjuvants as detailed in **Supplementary Methods**. At day 14, spleens were processed for *in vitro* testing.

### Cell Culture

Splenocytes from Tg/KO mice were cultured for 5 days in complete RPMI supplemented with 0.5% heat-inactivated, normal mouse serum (days 0-2) and 10% fetal bovine serum (days 3-5) in the absence or presence of 10 μg/ml of FLX-peptides or unmodified peptide. Secretion of IFN-γ was measured in cell culture supernatants by ELISA.

### Flow Cytometry

Flow cytometry was used for mouse phenotyping (**Figure S2**) and to analyze immune cell subsets by intracellular and/or surface marker staining as detailed in the **Supplementary Methods**.

## Results

### Effect of FLX Treatment on the Proteome and Immunopeptidome of B721-5701 Cells

**Figure 1:**
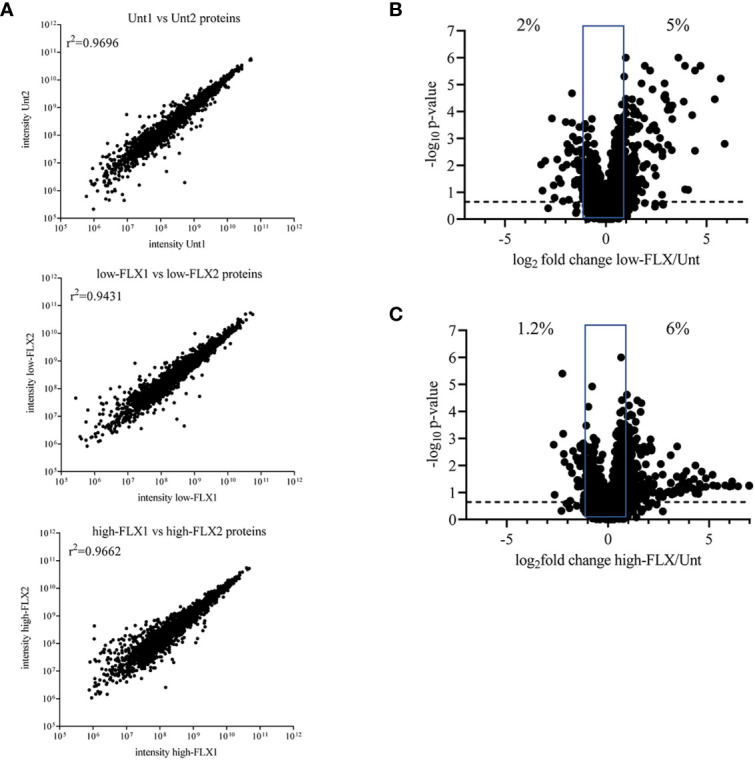
Flucloxacillin (FLX) does not significantly alter the B721-5701 cell proteome. Protein lysates were obtained from ten million B721-5701 cells treated with FLX at 150 μg/ml (low) or 453 μg/ml (high) for 5 days, or from untreated cultures (Unt), in duplicate. Samples were processed by LC-MS/MS and analyzed by Peaks 8.5 software. **(A)** Correlation among biological replicates. Protein abundance is represented in arbitrary units of intensity. **(B, C)** Increased (log_2_ fold change ≥ 1) and decreased (log_2_ fold change ≤ -1) abundance of proteins in the proteome of cells treated with low-FLX **(B)** or high-FLX concentration **(C)** compared to Unt, considering the average abundance of the two biological replicates per treatment. Percent values indicate divergence in abundance. Statistical differences were calculated by Spearman correlation. Proteome datasets are included in **Table S1**.

We hypothesized that FLX-driven changes in the protein content of the cell could impact the quality and quantity of antigen processing and presentation, including FLX-haptenated epitopes. B721-5701 cells were treated with subtoxic concentrations of FLX for 5 days (**Figure S3**). On average, 3,401 proteins were identified per sample by LC-MS/MS (**Figure 1A** and **Table S1**). A high Spearman correlation (>0.943) was obtained for all samples, independent of the treatment, indicating that overall, FLX treatment did not change the proteome of the cell. Changes in the relative counts (intensity) of <7% of proteins of the proteome of cells treated with drug vs untreated cells (**Figures 1B, C**) were not associated with any particular pathway when assessed by ingenuity pathway analysis (data not shown).

**Figure 2:**
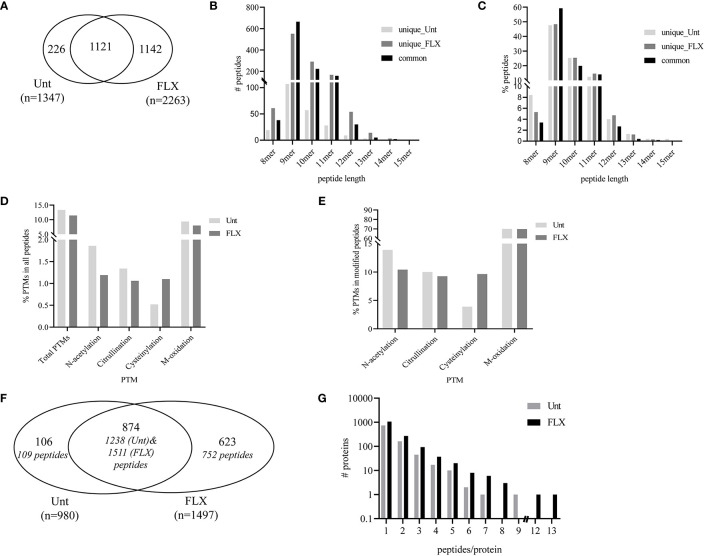
Flucloxacillin (FLX) treatment induces diversity in the peptide repertoire of B721-5701 cells. Common and unique peptides eluted from human leukocyte antigen (HLA)/peptide complexes obtained from two independent cultures of untreated B721-5701 cells (Unt) and of cells treated with 150 μg/ml of FLX (FLX) for 5 days were combined for the analysis **(A)**. Absolute counts **(B)** or frequency **(C)** of 8–15 amino acid peptides. Frequency of peptides with post-translational modification (PTM) within the peptide repertoire **(D)** or among modified peptides **(E)**. Common and unique proteins represented by immunopeptidome-contained sequences (in **Figure 2A**) **(F)**. Protein distribution showing the number of peptides per protein **(G)**. Data analysis was performed using Peaks 8.5 and X software. The datasets of peptides in the B721-5701 peptidomes are provided in **Table S2A, B**.

In parallel, cells were treated with FLX at 150 µg/ml for 5 days (FLX-cells) or left untreated (Unt-cells) in two biological replicates for immunopeptidome analysis. Among all four samples, LC-MS/MS analysis identified a total of 3,610 peptides of 8–15 amino acids in length, from which 1142 were detected only in FLX-cells and 226 peptides were only found in Unt-cells. The remaining 1121 peptides were common in both treatment groups (**Figure 2A** and **Tables S2A, B**). The peptide length distribution within each one of these subgroups was similar (**Figures 2B, C**), with dominance of sequences of 9–11 amino acids (89 ± 4%), from which 50% were 9-mers. Approximately 12% of the peptides in both datasets presented PTMs (**Figures 2D, E**). Cysteinylated peptides were twice as abundant in the FLX-cells as compared to the Unt-cells.

A total of 1,603 different protein sequences represented by the identified 3,610 peptides (**Tables S2A, B**). Of those, 874 proteins were identified in both treatment conditions, represented by 1238 peptides of the Unt-cells and 1511 peptides of FLX-cells peptide repertoire. The remaining protein sequences were unique to each treatment (**Figure 2F**). Overall, most of the proteins were represented by a single peptide (75.7% in Unt-cells and 70.8% in FLX-cells). Only 16.5% and 18% of the proteins were represented by two peptides (Unt-cells and FLX-cells, respectively), while 7.5% and 10% were proteins with three to five peptides (Unt-cells and FLX-cells, respectively) (**Figure 2G**). Of note, FLX-cells had a larger number of proteins with higher peptide representation than the Unt-cells.

**Figure 3:**
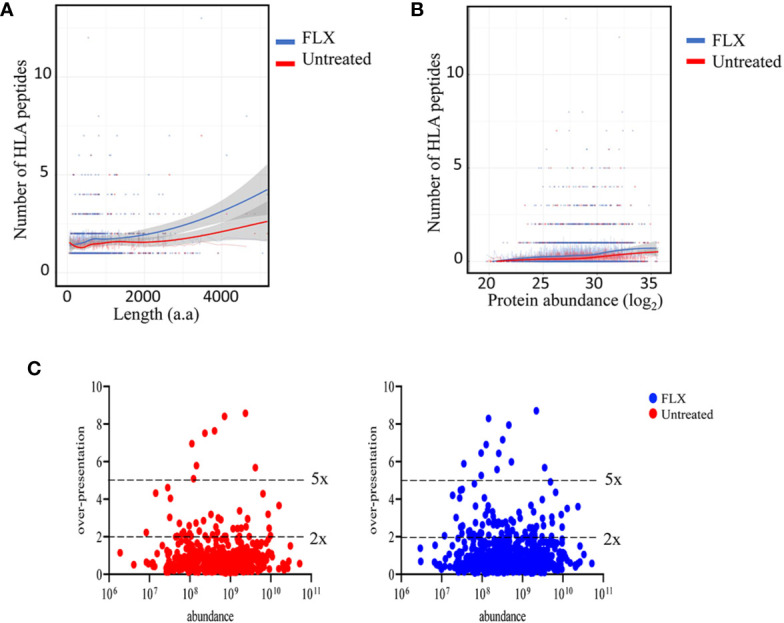
Flucloxacillin (FLX) treatment promotes a higher peptide sampling rate of proteins, independently of the length and abundance of the polypeptide. Abundance of human leukocyte antigen (HLA) peptides from 435 and 699 proteins in the proteome of B721-5701 cells untreated or FLX-treated, respectively, as a function of the protein amino acid length **(A)** or abundance (log_2_) **(B)**. Individual proteins and trendlines are represented in the graphs. **(C)** Protein fold over-presentation was calculated based on the ratio between observed (D) and expected (D’) peptide density, and represented as function of protein abundance. The dataset of the represented proteins is provided in **Table S3**.

In agreement with others (19, 20), only a fraction of the identified proteins in the proteome were represented in the HLA peptidome of Unt-cells (13%) and FLX-cells (21.8%), corresponding to a total of 435 and 699 proteins, respectively. As expected, longer or more abundant proteins contributed to more peptides in the immunopeptidome (**Figures 3A, B**), although the peptide number/protein was significantly higher in both sampled unique and common proteins of FLX-cells (**Figure S4** and **Table S3**). We then calculated the HLA class-I sampling density (D) as described previously (19, 20), a normalization score that takes into account the number of observed HLA peptides of a given protein as a function of its length, and used D to identify proteins that were presented at a higher rate than what was expected for their abundance (represented by D’). Most of the proteins with high over-presentation scores (D/D’>3) had roles in transcription, translation or mitochondrial-related pathways (**Table S3**). Ribosomal subunits had the highest scores (D/D’>5) in both treatment conditions. Interestingly, β_2_-microglobulin epitopes were found in the FLX-cell immunopeptidome but not in the Unt-cell (D/D’>6). In general, the FLX-cell proteome had more over-presented proteins (D/D’>2) than the Unt-cell proteome (86 vs 47, respectively) (**Figure 3C** and **Table S3**). This trend did not correlate with protein abundance (r^2^<0.00230 and 0.00196 for the control and FLX-treated group, respectively) (**Figure 3C**). These results suggest a qualitative and quantitative impact of the drug in the cell immunopeptidome, which is not associated with a change of the cellular proteome but most likely to an effect on antigen processing and presentation.

### FLX Treatment Increases HLA-B*57:01-Presented Peptides Containing Tryptophan in the PΩ Position

**Figure 4:**
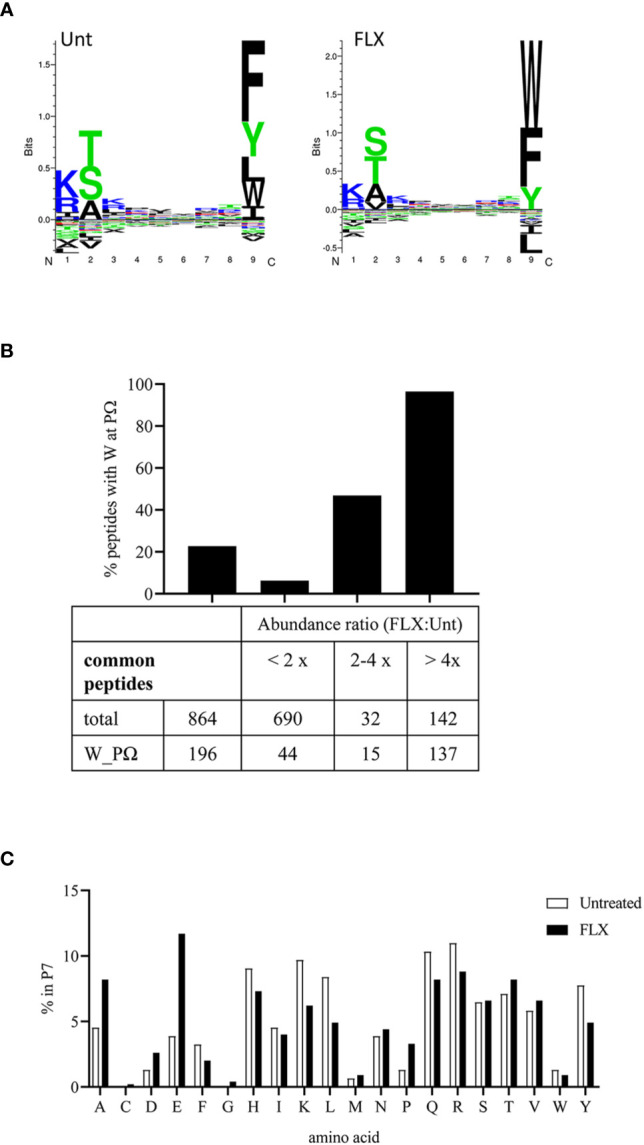
Flucloxacillin (FLX) treatment does not alter consensus peptide binding motif of HLA-B*57:01 peptides but favors peptides with tryptophan in P9/PΩ. **(A)** Unsupervised analysis of the sequence motif from 9-mer sequences in the immunopeptidome of B721-5701 cells, untreated (Unt) or treated with FLX for 5 days (FLX), using Gibbs Cluster 2.0 software (peptide sequence details provided in Table S2A-B). Peptides with PTMs were excluded from this analysis. **(B)** Ratio of the abundance of the common peptides in the FLX vs Unt immunopeptidome samples, including total and C-terminal tryptophan (W) peptides. Ratios are represented as FLX : Unt lower than two-fold, between two- and four-fold or higher than four-fold. Abundance values were calculated using Quant analysis of common peptide quantifiable IDs (<1% FDR). **(C)** Frequency of the different amino acid residues at P7 of 9-mers with predicted binding affinity <50 nM (by NetMHC 4.0 software).

The peptide repertoire presented by HLA-B*57:01 molecules is determined by the presence of specific amino acids in the anchor positions of its binding motif. Unlike peptides identified from abacavir-treated cells (13–15), 9-mers in the immunopeptidome of FLX-cells exhibit consensus amino acids for the HLA-B*57:01 peptide anchor motif at position P2 and P9 (**Figure 4A**). However, FLX-cells presented a higher frequency of peptides with tryptophan at PΩ than those untreated, mainly in unique peptides (3.7% in Unt *vs* 79.8% in FLX) (**Tables S2A, B** and **Figure 4A** for 9-mers). Common peptides with C-terminal tryptophan were also more abundant in the FLX-cells (**Figure 4B**). This phenomenon was also observed in two additional independent experiments (**Figure S5**). Interestingly, peptides with C-terminal tryptophan had the highest predicted HLA-binding affinity (<50 nM). Although arginine was dominant at P7 [a secondary anchor residue (21)] of high-affinity peptides, glutamic acid was significantly increased in peptides of FLX-cells (**Figure 4C** and **Tables S2A, B**). These findings suggest that, in contrast to the ability of abacavir to alter the anchor motif of the neoepitopes from phenylalanine, tryptophan or tyrosine to leucine or isoleucine at PΩ, FLX increases the presentation of self-epitopes with predominantly tryptophan at the C-terminal position.

### Identification of FLX-Haptenated Self-Peptides Derived From sHLA in B721-5701 Cells

**Figure 5:**
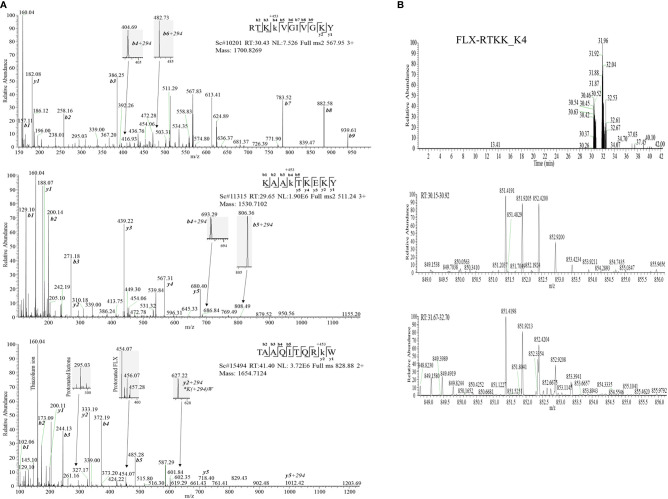
Flucloxacillin modified peptides identified from drug treated HLA-B*57:01 expressing cells. **(A)** MS/MS spectra of parent ions with characteristic b and y ions, drug fragments (160.04, 295.03, 454.06/07) and diagnostic b+294 fragments identified: RTKK(FLX)VGIVGKY (FLX-RTKK_K4, top panel), KAAK(FLX)LKEKY (FLX-KAA_K4, middle panel), and FLX-modified TAAQITQRK(FLX)W (FLX-TAA_K9, bottom panel). **(B)** Two FLX-RTKK_K4 isoforms, with different retention time by RP-HPLC (peaks at minute 30.46 and 31.92) (top chromatogram) but identical mass (m/z=851.4191/2+, m/z=567.85/3+ (data not shown)) (mid and low LC-MS1 spectra). All peptides were identified using PEAKS analysis and verified manually.

Drug conjugation to self-peptides has been proposed as a molecular mechansim by which FLX leads to the generation of neoepitopes and T cell reactivity (4, 5, 12). Thus, we focused on identifying FLX-haptenated peptides (FLX-peptides) in our peptide dataset. Initial experiments with small scale cultures did not identify FLX-peptides, suggesting that haptenated peptides were in low concentration. In an attempt to increase the amount of HLA and thus the frequency of FLX-sequences, we cultured B721-5701 producing a soluble form of HLA (sHLA) in high density biopharms as described previously (14). We identified several peptides containing FLX on lysines (K) as well as the corresponding unmodified sequences: TAAQITQRKW (TAA) from HLA, KAAKLKEKY (KAA) from the high mobility group HMGB-1 protein and RTKKVGIVGKY (RTKK) from a 60S ribosomal protein. The latter two peptides revealed multiple possible lysine-drug conjugation sites (**Table 1**). Collision-induced dissociation MS2 resulted in complex spectra with peptide fragments along with drug ions +160, +295, and +454 as labelled on the TAA peptide spectrum in **Figure 5A**, even though intact drug was not found on fragmented sequences. However, drug-modified peptides did retain diagnostic fragments containing K+294 FLX, the residual portion of FLX covalently linked to the peptides as illustrated in **Figure 5A**. We identified b4-K+294 in FLX-RTKK_K4 and FLX-KAA-K4, and y2-K+294 in FLX-TAA_K9. Interestingly, the modified lysine in TAA corresponded to K146 of the HLA, an interaction site with KIR-3DL immuno-regulatory receptor of NK cells. Peptides were further validated by chemical syntheses using FLX-coupled Fmoc lysines as described in Methods (**Figure S6A**). In addition, we synthesized two KAA peptides with FLX in lysine 6 or 8 (FLX-KAA_K6 and FLX-KAA_K8) and RTKK with drug in the lysine in position 10 (FLX-RTKK_K10), as controls for immunogenicity studies, although these were not identified in the peptide repertoire. Quality control runs of the synthesized FLX-peptides surprisingly showed RP-HPLC chromatograms with two peaks by retention time (RT) each with the same mass by LC-MS (**Figures S6B, C**). FLX-RTKK_K4 isomeric forms differing in RT were later confirmed in the B721-5701 immunopeptidome (**Figure 5B**). We hypothesized that the difference in RP-HPLC RT was secondary to conformational differences between the FLX adducts. For these reasons, the two RP-HPLC peaks of the FLX-RTKK peptides were purified separately and tested independently in *in vivo* experiments.

**Table 1:** Unmodified and flucloxacillin (FLX)-modified peptides found in B721-5701_HLA cell preparations.

Peptide	FLX-conjugated residue in the protein	Peptide Length	Mass	m/z	z	Protein Accession	Protein Description
KAAKLKEKY KAAK(+453.06)LKEKY	na K150	9 9	1,077.6545 1,530.7102	539.8342 511.2447	2 3	P09429|HMGB1_HUMAN	High mobility group protein B1
RTKKVGIVGKY RTKK(+453.06)VGIVGKY	na K7	11 11	1,247.7714 1,700.8269	624.897 567.9514	2 3	P61513|RL37A_HUMAN	60S ribosomal protein L37a
TAAQITQRKW TAAQITQRK(+453.06)W	na K145	10 10	1,201.6524 1,654.7124	602.34 828.3697	2 2	P18465|1B57_HUMAN	HLA class I histocompatibility antigen B-57 alpha chain

na, not applicable.

### The Immunogenic Potential of FLX-Peptides Depends on the Peptide Sequence and the Drug-Conjugated Amino Acid Position

**Figure 6:**
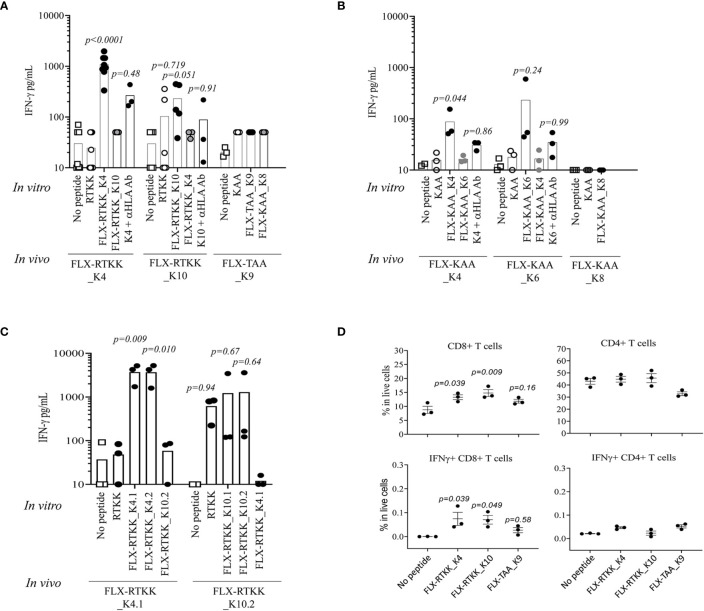
Immunogenicity potential of flucloxacillin (FLX)-haptenated peptide is dependent on the peptide sequence, the position of the lysine to which drug is conjugated and the expression of human leukocyte antigen (HLA). **(A, B)** Tg/KO mice (n=3–9) were immunized by 2 s.c. doses of 100 μg of FLX-peptides together with 150 μg of HBV_128-140_ CD4^+^ T helper peptide with IFA. Splenocytes were subsequently stimulated *in vitro* with 10 μg/ml peptides (as indicated) for 5 days. IFN-γ was measured in the supernatant of the cultures by ELISA. *In vitro* responses to the *in vivo* immunogen were blocked by anti-HLA B/C antibody. **(C)** Immunizations and cultures were set up similarly to that described in A but with peptide isoforms of the FLX-RTKK peptides. **(D)** IFN-γ intracellular staining of day 5 splenic cell cultures from animals treated with FLX-peptides and stimulated *in vitro* with the same peptide. Graphs show results gated in CD8^+^ or CD4^+^ T cell populations (one representative of two experiments). Statistical analysis by one-way ANOVA using the no peptide condition as baseline. *p-values* are only shown if <0.999.

To evaluate the immunogenicity potential of the FLX-peptides, we immunized Tg mice expressing only the chimeric HLA-B*57:01/H2-D^d^(α3) and not classical mouse MHC-class I (H2-K^b^ and H2-D^b^) molecules (Tg/KO). Peptide-specific T cell responses were observed in animals treated with several FLX-peptides upon splenocyte *in vitro* restimulation (**Figures 6A, B**). FLX-RTKK_K4 induced higher levels of IFN-γ secretion than FLX-RTKK_K10 in cells of animals immunized with the same peptides, and did not cross-react with either the parent, unlike cells from some of the FLX-RTKK_K10-immunized mice, or the other modified counterpart. (**Figures 6A, C**). Both FLX-RTKK peptide isomers showed similar specificity and immunogenic potential (**Figure 6C**). The IFN-γ response was driven by CD8^+^ but not CD4^+^ T cells (**Figure 6D**). Importantly, the recognition of FLX-peptides by CD8^+^ T cells was dependent on the HLA-B*57:01 expression, as evidenced by the effect of an anti-HLA antibody on IFN-γ secretion (**Figure 6A**). Immunizations with FLX-KAA_K4 and _K6 also generated antigen-specific and HLA-dependent T cell responses but at lower magnitude than the FLX-RTKK_K4. Control FLX-KAA_K8 showed no T cell reactivity (**Figure 6B**). FLX-TAA_K9 also failed to induce an immune response (**Figures 6A, D**). These results suggest that peptides bearing modified lysines in specific positions are more immunogenic than others depending on their exposure to solvent and accessibility to TCR.

**Figure 7:**
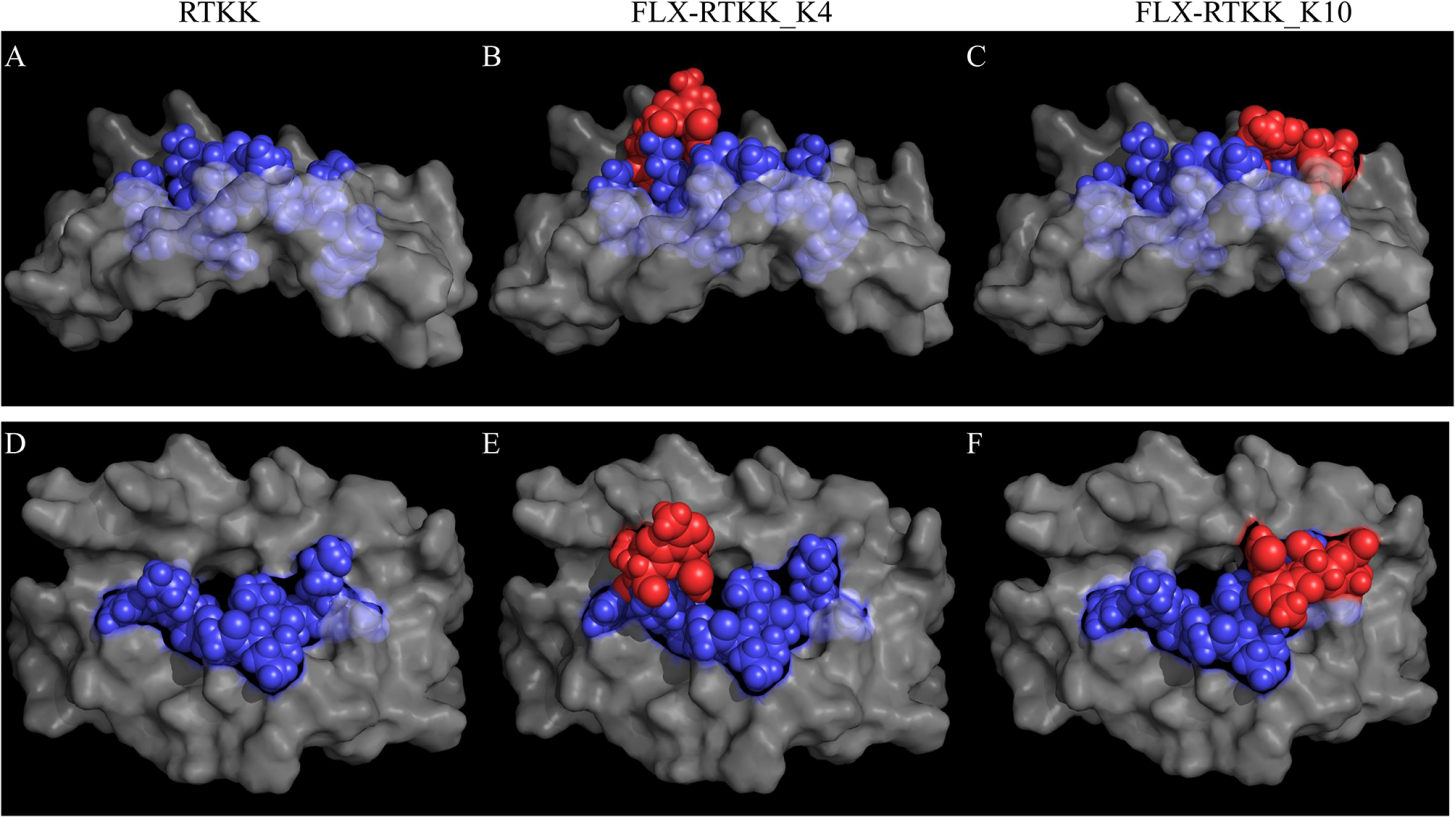
*In silico* prediction of the interactions of parent and flucloxacillin (FLX)-RTKK peptides with HLA-B*57:01 cleft. KAF11 peptide residues (PDB ID: 2YPK) were replaced with those of the RTKK peptide. Docking the peptide into the HLA cleft was performed using FlexPepDock and addition of the drug to the appropriate lysine and energy minimization of the structure using OpenBable (v3.0.0) software. Structures were visualized in PyMOL (v2.3.4). Lateral **(A–C)** and top **(D–F)** views of the human leukocyte antigen (HLA) complex with the unmodified parent sequence of RTKK **(A, D)**, FLX-RTKK_K4 **(B, E)** and FLX-RTKK_K10 **(C, F)**. Peptide sequences are represented in blue spheres, drug is represented by red spheres and α1α2 domains of the HLA are depicted in gray color.

To visualize the spatial position of the drug on the peptide, we used computational modeling. This was achieved by docking the peptide into the HLA cleft using FlexPepDock software (22, 23), followed by addition of the drug to the appropriate lysine and energy minimization of the structure using OpenBabel (24). Differences in the location of the drug on the HLA/peptide complex were evident between the immunogenic and not immunogenic peptides (**Figure 7** and **Figure S7**). FLX in lysines at P4 (i.e. FLX-RTKK_K4 in **Figures 7B, E**) or P6 (FLX-KAA_K6 in **Figures S7C, G**) occupies a central location on the HLA cleft, resembling the bulge of a longer peptide, whereas when conjugated in PΩ-1, (i.e. FLX-RTKK_K10 in **Figures 7C, F**) it occupies a position on the outer border of the HLA-bound peptide. Although we do not have FLX-specific T cell receptors (TCR) to model this interaction, we superposed a TCR from the HIV KAF TCR (2YPL) on FLX-RTKK_K4 and _K10 (**Figure S8**). The TCR footprint is consistent with FLX-peptide recognition at K4 interacting with the TCR V-alpha CDR3 (**Figure S8A**). This contrasts with peptide conjugated FLX at PΩ-1 that is oriented more towards TCR V-beta CDR3 (**Figure S8B**).

Althogether, these data indicate that FLX-peptides are immunogenic in association with HLA-B*57:01 and the potential of FLX to activate a CD8+ T cell response is dependent on the position of the lysine residue in the peptide sequence.

## Discussion

Idiosyncratic FLX hypersensitivity reactions driven by immune cells occur at low frequency (2, 25, 26). Expression of HLA-B*57:01 and HLA-B*57:03 alleles was recognized as genetic risk factors for FLX-induced liver injury (6, 7), and thus attention has been paid to understand the molecular mechanisms by which drug can generate neoepitopes that are presented by HLA to activate T cells. To examine these mechanisms, we characterized the effect of FLX on the cell proteome and immunoproteome looking for gobal changes as well as appearance of drug-haptenated peptides.

Alterations of the B721-5701 cellular protein pathways by FLX could impact the abundance of self-epitopes and PTMs as well as the generation of neoepitopes, using these potential mechanisms to boost protein immunogenicity. Although no significant changes were observed in the total protein content of the cells when incubated with FLX, drug treatment induced a higher diversity in the peptide repertoire presented by the HLA which could impact antigen presentation. Increased abundance and diversitiy of sequences of particular proteins in MHC complexes can be due to an enhanced processing rate of such proteins (19). Consistent with this hypothesis, we observed a larger number of proteins represented in the FLX-immunopeptidome with peptide presentation rate above those predicted considering the protein length. Alteration of the peptide processing (immunoproteasome *vs* proteasome) or the peptide loading (e.g. tapasin-dependent or -independent mechanisms) machineries could explain qualitative and quantitative changes on the cell immunopeptidome. Penicillinase-resistant antibiotics, including FLX can induce endoplasmic reticulum stress, which can lead to misfolding of proteins, oxidative stress and caspase-3 activation (27). Although the drug concentrations used in our study were lower than in those studies (0.3-1 mM *vs >*16mM) and the cells tested were different (B cells *vs* hepatocytes), we found that caspase-3 protein expression was elevated in our 1mM-treated FLX-cells, and thus, could indicate the occurrence of non-cytotoxic changes impacting protein processing. However, the mechanisms by which FLX may impact proteosome processing and/or peptide loading and presentation without changing the global protein content of the cell requires further study (see further discussion below).

The generation of neoepitopes by drugs inducing immune-mediated hypersensitivity reactions is believed to occur by covalent adduct formation, pharmacological interaction of the drug with the peptide, and non-covalent interaction leading to alteration of the peptide repertoire (28) which most likely coexist *in vivo* (29). In our study, no changes were observed in the consensus anchor residues of peptides isolated from FLX-cells, either at the P2 or PΩ positions, consistently with other reports (13–15, 30, 31) A lower frequency of tryptophan at P9 was observed in some of our untreated preparations (**Figure S5**) contrasting with what others reported on HLA-B*57:01 peptide repertoires (21, 31). This variation could be attributed to differences in culture conditions such as absence of other β-lactam antibiotics in the media other than FLX or the use of different cell lines. Nevertheless, and unlike abacavir that increases the presentation of epitopes with unconventional isoleucine or leucine residues in PΩ (13–15), FLX promoted an enrichment of tryptophan at PΩ, in agreement with a trend observed in a recent report (31). We established that the overall net higher frequency of peptides with tryptophan at PΩ in the FLX-cells included both unique sequences (qualitative) and higher abundance in common peptides (quantitative). Because of its greater size, it seems unlikely that FLX can fit directly into the HLA F-pocket in a manner similar to that of abacavir. Therefore, we hypothesize that changes in the peptide repertoire may result from an effect on the peptide processing machinery. Catalytic shifts in proteasomic subunits induced by pathogenic infections but also by oxidative stress (32) increase the immunoproteosome activity of the cell resulting in an enrichment of peptides with higher content of hydrophobic amino acids at the C-terminus promoted by the chymotrypsin activity of LMP2 (33), perhaps favoring tryptophan (34). Whether this occurs indirectly or by direct interaction of FLX with one of the immunoproteasome complex subunits, such as LMP2 or LMP7, has not been addressed in our study. In our studies, peptides with tryptophan in the C-terminus have higher predicted binding affinity to HLA-B*57 (**Tables S2A, B**), consistent with other findings (21). Loading of high-affinity peptides by tapasin-dependent mechanisms could impact self-tolerance, possibly contributing to drug hypersensitivity such as in cases where tissue damage continues even after discontinuation of drug administration.

T-cell recognition of FLX by cells of healthy individuals carrying HLA-B*57:01 can involve processing dependent and independent pathways (4, 5, 8). However, the activation of CD8^+^ T cells from HLA-B*57:01^+^ DILI patients by FLX is peptide processing dependent and restricted to the expression of B*57:01 allele (4, 8) possibly explaining the low frequency of FLX-associated DILI and why only the expression of the HLA-B*57:01 or B*57:03 alleles has been linked to FLX-induced liver injury. Processing dependent T cell activation suggests that covalent modifications of protein epitopes generate neoantigen drug targets. To study the immunogenic potential of FLX-conjugated peptides, we used mass spectrometry analysis of HLA purified peptides and identified three FLX-peptide sequences in the B721-5701 immunopeptidome of drug-treated cells. One peptide FLX-TAA_K9 was from HLA itself suggesting that lysines on the HLA surface may also be targets for direct drug modification (31), analogous to FLX modifications of lysines on albumin (12). The other two peptides had multiple lysine residues and were from internal proteins, KAA from a nuclear HMGB-1 DNA binding protein and RTKK from the 60S ribosomal protein, both of which are in high abundance in the cell. Both peptides were modifed on the P4 lysines, suggesting a preference at this position on presented peptides. Generation of haptenated peptides could occur either on the intact protein or on free peptide after proteosome cleavage, as suggested in a recent report by Waddington et al. (31) who also identified FLX modifications on TAA and RTKK, among other peptides. In their study, the position of the modified amino acid in peptides such RTKK was ambiguous and not verified by synthetic chemistry.

Using FLX-lysine Fmoc synthetic conjugates, we synthesized both FLX-peptides identified in the cell immunopeptidome as well as other analogs with FLX on other lysine residues to confirm the identity of the sequences, the position of the drug, and to assess their immunogenic potential Interestingly, RP-HPLC profiles of the synthesized FLX-peptides as well as FLX-RTKK_4 isolated from the cells showed two peaks with distinct retention times but with exact mass, unlike those of the unmodified sequences. We believe that these differences represented two conformational states of the open β-lactam ring of the antibiotic, since two different peaks were also observed for the Fmoc FLX-lysine conjugate after deprotection but not for the FLX-lysine with the intact β-lactam ring (data not shown). Further structural work is required on FLX-peptide conjugates to define these properties and to study further its relation to peptide immunogenicity.

To estimate the immunogenicity potential of FLX-epitope sequences, we immunized HLA-B*57:01 mice lacking the expression of the murine MHC-I molecules H-2K^b^ and H-2D^b^ with the selected FLX-peptides. Different levels of immunogenicity were observed depending on the peptide sequence and the position at which the drug was conjugated. Cells from animals primed with FLX-RTKK_K4 or FLX-KAA_K4 sequences, unlike modified TAA peptide, were quickly activated *in vitro* to produce IFN-γ in an antigen-specific and HLA-dependent manner. Interstingly, peptides not detected in the immunopepditome like FLX-KAA_K6 and FLX-RTKK-K10 showed increased immunogenicity (the latter with less specificity), while FLX-KAA_K8 did not activate T cells. For FLX-RTKK peptides, the presumably isomeric sequences were recognized similarly by primed T cells. Failure to identify weak immunogenic peptides in the cell immunopeptidome could be explained by a disadvantage of certain lysine residues to be modified by the drug or interference in peptide loading. Both the physicochemical properties of the peptide (35) and the contact potential among TCR and peptide (36) have been postulated as critical parameters in the prediction of epitope immunogenicity. Notably, FLX conjugates were not found in anchor residues (P2 or PΩ) and the lysine side-chains of FLX-peptides were oriented exposed to solvent away from the HLA cleft. Interestingly, 3 out of the 4 immunogenic peptides tested had drug in lysines in position P4 or P6, positions that are considered critical for contact specificity and generation of immunogenicity (35). Structural models presented here for P4 and P6 suggest that drug modifications in these positions could be seen by T cells in a manner similar to large peptides that contain bulges in central regions. Using the HLA-B*57:01 restricted HIV-KAF TCR as a model, we showed that the lysine at P4 is at a site that overlaps with the hypothetical footprint of TCR V-alpha CDR3 while lysines at PΩ-1 are not, suggesting that TCR chains could be differentially selected specifically based on the amino acid position of the drug linkage. FLX at PΩ-1 may be outside the footprint of the CDR3 chains or have difficulty in peptide processing/loading to HLA.

As mentioned above, FLX-TAA_K9 is derived from HLA-B*57:01 itself. Direct modification of lysines on HLA has intriguing implications in addition to being target epitopes for T cells after antigen processing. HLA-K146, corresponding to the FLX-modified lysine in FLX-TAA_K9, is positioned near the HLA F-pocket where the peptide C-terminus will interact and is highly conserved among HLA molecules. K-146 is critical for HLA binding to the inhibitory KIR-3DL1 protein on NK cells (37, 38). Direct modification of HLA-K146 by FLX as modeled in **Figure S9** could interfere with the KIR-3DL1-binding and possibly lead to NK activation. Similarly, FLX modification on lysines positioned at PΩ-1 such as on FLX-TAA_K9 could also interfere with the KIR3DL1-binding site and impact KIR function as KIR3DL1 binding was reported to depend on the nature of the amino acid at the PΩ-1 (P8 in Vivian et al. (37)). Activation of NK in addition to CD8^+^ T cells have been reported in PBMCs isolated from patients with drug hypersensitivity reactions (39) as well as in fluid of skin blisters of patients with Stevens-Johnson syndrom (40), although the exact molecular mechanism involved in such activation is yet to be determined. Further studies are required to elucidate whether K146 or other FLX-modified lysines on HLA can interfere with KIR or T cell receptor binding.

In summary, factors leading to idiosyncratic drug hypersensitivity might involve non-covalent and covalent interactions between drug and peptide. In our study, we showed that FLX treatment of B721-5701 cells can alter the presentation of self-peptides by enriching the peptide repertoire of the cell with sequences containing tryptophan at the C-terminus, a preferred amino acid for immunoproteasome processing and which confers a higher predicted binding affinity to the sequence. We also identified drug-haptenated peptides with immunogenicity potential depending on the lysine at which FLX gets conjugated. Direct modifications of the HLA by FLX at lysines that are believed to play immunoregulatory roles could also participate in the activation of innate immune pathways, contributing inflammatory events to the initiation of immune-mediated drug adverse reactions. Further animal studies will allow us to characterize the generated immune responses in more detail and whether the reactive cells can infiltrate hepatic tissue and lead to DILI. Understanding the mechanisms behind these modifications and their impact on breaking T cell tolerance, individually or as a whole, is critical to drug development and amelioration of adverse side effects.

## Data Availability Statement

The raw data supporting the conclusions of this article will be made available by the authors, without undue reservation.

## Ethics Statement

The animal study was reviewed and approved by Institutional Animal Care and Use Committee at FDA.

## Author Contributions

MP and MN conceived, planned, and supervised the experiments. MP, SA, RV, SP, EM, MC, SS, and GR performed the experiments. MT, SB, and GA chemically synthesized the peptides. WW performed mass spectrometry analysis. LW and ZL performed proteomics data analysis. WH generated large-scale HLA preparations. LB, KN, and DM generated the HLA-B57*01 Tg/KO mice. MP, DM, and MN edited and reviewed the manuscript. All authors contributed to the article and approved the submitted version.

## Funding

This work was supported by the FDA Intramural Research Program, and in part by a Senior Postgraduate Research Fellowship Award to S.P., E.M. and S.S. from the Oak Ridge Institute for Science and Education (ORISE) through an interagency agreement between the U.S. Department of Energy and the U.S. Food and Drug Administration. DHM’s group is supported by the Intramural Research program of the NIAID, NIH.

## Conflict of Interest

The authors declare that the research was conducted in the absence of any commercial or financial relationships that could be construed as a potential conflict of interest.
