# Supplementary material for: Alterations in the HLA-B*57:01 Immunopeptidome by Flucloxacillin and Immunogenicity of Drug-Haptenated Peptides
Source: Front Immunol. 2021 Feb 9;11:629399. doi: 10.3389/fimmu.2020.629399 (PMC7900192; doi:10.3389/fimmu.2020.629399)
Supplement: Supplementary file 1 [file DataSheet_1.docx]

**SUPPLEMENTARY MATERIALS and METHODS:**

**Reagents**:

Flucloxacillin (FLX) was obtained from Apotex, UK or Sigma-Aldrich, USA. FLX-conjugated lysine residues were prepared in house following Scornet et al’s method (1) and drug-conjugation was confirmed by nuclear magnetic resonance (data not shown). For details, see the section on Synthesis of FLX-Lysine and Suppl Fig 1. Drug-modified peptide sequences were synthesized by solid-phase Fmoc chemistry on a Tribute^R^ peptide synthesizer (Protein Technologies, Inc., Tucson, Arizona) using the FLX-conjugated lysine residues. Subsequently, peptides were HPLC purified on an XBridge BEH C18 OBD Prep column (Waters, Milford, MA), quantified and lyophilized.

**B721-5701 protein lysate and LC-MS/MS analysis:**

B721-5701 cells were grown in cell culture flasks for 5 days to a density of 1-2 million cells/mL in R-10 medium (RPMI (Gibco BRL) containing 10% fetal bovine serum (Atlanta Biologicals), 7.5 mM HEPES (MP Biomedicals), 2 mM L-glutamine (MP Biomedicals), and 150 µM non-essential amino acids (Gibco BRL), in absence or presence of FLX (150 or 453 μg/mL), in duplicate. Cells (10-12 million) were washed in 1x PBS and resuspended in RIPA Lysis Buffer containing protease inhibitors, freeze/thawed 3 times and sonicated (Branson Digital Sonifier) to obtain a cell lysate. The protein extract was clarified by spinning the sample at 14,000 *xg* for 15 minutes at 4 ºC and its protein concentration was determined by the Pierce^TM^ bicinchoninic acid (BCA) method. Aliquots were stored at -20 °C until further use. One milligram of protein extract was treated with RapiGest surfactant (0.1%) for 30 min at 45 °C and 500 rpm. Subsequently, samples were treated with 50 μL of 50 mM ammonium bicarbonate for 15 min at 37°C, followed by 50 μL of 10 mM DTT at 80°C with shaking for 15 min, and followed by 50 μL of 20 mM iodoacetamide at 37°C for 30 min, spinning in between steps to discard the liquid. Samples were further digested with trypsin at 20:1 protein:protease ratio (w/w) and incubate overnight at 37 °C. Trifluoroacetic acid was added to 0.5-1.0% (v/v) to stop the digestion by reducing the sample pH to 2 and samples were incubated at 37 °C for 10 min.

The protein digest (600 μg) was used for strong-cation exchange pre-fractionation using PolySULFOETHYL A column 200 A x 20 cm with particle size 5 μm (PolyLC, Columbia, MD) using the mobile phase A: 5mM KH2PO4, pH 2.7 and 30% ACN, and mobile phase B: 5mM KH2PO4 in 350 mM KCl, pH 2.7 and 30% ACN and flow rate 200 μL/min. The gradient starts from 0% B for 10 mins, 26% B by 15 mins, 35% B by 40 mins, 60% B by 45 mins, 100% B by 49 mins. After 55 mins column comes to the initial washing conditions for 9 mins at 0% B. Each sample was collected into 9 fractions at 0-10 mins, 10-20 min, 20-22.5 min, 22.5-25 min, 25-27.5 min, 27.5-30 min, 30-40 min, 40-55 min, and 55-64 min.

SCX- fractions were lyophilized and dissolved in 20 μL of 0.1% formic acid buffer for LC-MS experiments. Thermo Q-Exactive Orbitrap mass spectrometer coupled with Eksigent nanoflex cHiPLC system was used for the proteomic experiments. Briefly, proteomic fractions were loaded on trap column and switched onto C18-analytical column at 2% B (0.1% formic acid in acetonitrile) for 2 mins. Peptides eluted linearly over 120 mins of time to reach 30% B. After, column was equilibrated with 2% B for 15 mins. The flow rate was maintained at 250 nL/min. The Q-Exactive mass spectrometry conditions are full MS spectra recorded at 70,000 resolution, scan range 400-1200 m/z, AGC target 3e6, and maximum injection time (IT) of 250 ms. Subsequently top 10 ions were submitted for high collision-energy dissociation (HCD MS/MS) using stepped collision energy 25, 27, 30 eV at 17,500 resolution, AGC target 2e5, isolation window 2 m/z and maximum IT 120 ms with fixed first mass 100 m/z. Dynamic exclusion was set up for 15 secs. LC-MS/MS readouts were done in triplicate for each fraction.

LC-MS/MS derived .raw files were submitted for PEAKS 8.5 analysis using the Uniprot non-redundant human database. Data was analyzed at 10 ppm for MS1 analysis and 0.05 Da for MS2. We included trypsin as enzyme, carbamidomethylation as the fixed modification and variable modifications such as N-terminal acetylation of peptide, oxidation of methionine (M), deamidation of asparagine (N) and glutamine (Q). The allowable maximum missed cleavages and modifications per peptide are 2.

**Generation and isolation of HLA-bound peptides:**

***• Production of mHLA/peptide complexes and peptide purification:*** B721.5701 cells were grown in G-Rex 6 Well Plates (Cat. # 80240M, Wilson wolf, New Brighton, MN) seeded at 10^7^ cells per well in 30 mL of medium (RPMI containing 10% fetal bovine serum, 2 mM L-glutamine, 1 mM sodium pyruvate (MP Biomedicals)), in the presence or absence of FLX (150 μg/mL) for 5 days and grown to approximately 2x10^8^ cells. Pelleted cells were lysed with 2.5 ml of lysis buffer (consisting on 2 mM EDTA, 2 mM PMSF, Protease inhibitor cocktail (Roche), 0.5% Sodium Deoxycholate, 2% Octyl-β-D glucapyranoside, dissolved in PBS) on ice for ten minutes. Aliquots of 0.5 mL of the cell lysate were subsequently sheared with a mechanical homogenizer and clarified by spinning it down at 4 ^0^C at 14000 rpm for 50 minutes. Pooled lysate supernatants were immunoprecipitated with HLA I (W6/32) antibody crosslinked with Protein A overnight at 4 ^0^C with gentle rocking. Peptide elution was performed according to Chong et al method (2) using a Waters Positive Pressure-96 Processor (Waters, Milford, MA). Briefly, 96-well single-use micro-plate with 3 μm glass fiber and 10 μm polypropylene membranes (204495-100 Agilent) were washed and equilibrated with 1 mL of 100% acetonitrile (ACN), followed by 1 mL of 0.1% TFA and lastly with 2 ml of 0.1 m Tris-hydrochloric acid (pH 8.0). The immunoprecipitated lysate was subsequently added to the plate, using four wells per sample. Samples were washed consecutively 4 times with each one of the following buffers (2 mL per wash): buffer 1 (consisting of 0.005% IGEPAL 630, 50 mM Tris pH 8.0, 150 mM NaCl, 5 mM EDTA, 100 µM PMSF and 1 µg/mL Pepstatin A); buffer 2 (consisting of 50 mM Tris pH 8.0 and 150 mM NaCl); buffer 3 (consisting of 50 mM Tris pH 8.0 and 450 mM NaCl); buffer 4 (50 mM Tris pH 8.0). Addition of 500 μL of 1% TFA assisted in transferring the samples onto a C18 96-well plate (ref number: 186002321, Waters), previously conditioned with 1 mL of 80% ACN in 0.1% trifluoroacetic acid (TFA) followed by 2 mL of 0.1% TFA. Upon an additional wash with 2 mL of 0.1% TFA, peptides were eluted from the HLA complexes with 500 μL of 28% ACN in 0.1% TFA. Peptides were pooled from all 4 wells, vacuum-dried and dissolved in 25 μL of 0.1% TFA. Samples were analyzed by LC/MS/MS using a ThermoFisher Ultimate LC and Fusion Orbitrap MS (San Jose, CA). Briefly, peptides were first loaded onto a trap cartridge (ThermoFisher PepMap, C18, 5 µm, 0.3x5 mm), then eluted onto a reversed phase Easy-Spray column (ThermoFisher PepMap, C18, 3 µm, 100A) using a linear 120-min gradient of acetonitrile (2-40%) containing 0.1% formic acid at 250 nL/min flowrate. The eluted peptides were sprayed into the Fusion Orbitrap. The data-dependent acquisition (DDA) mode was enabled, and each FTMS MS1 scan (60,000 resolution) was followed by FTMS MS2 scans (15,000 resolution) using top speed (acquire as many MS2 scans as possible within three second cycle time). Precursor ion (selected at 1.6 Da window) fragmentation took place at HCD cell with CE of 30. AGC targets are 2.0e5 and 5.0e4, respective, for MS1 and MS2. The spray voltage and ion transfer tube temperature were set at 1.8 kV and 250 ºC, respectively.

***• Production of sHLA/peptide complexes and peptide purification:*** sHLA from FLX-treated cultures was collected from continuous cultures as described previously (3). Briefly, B721-5701 cell transfectants were cultured in high density hollow fiber bioreactors. Milligram quantities of sHLA-peptide complexes were purified from pooled supernatants from continuous cultures using affinity chromatography W6/32 columns. Peptides were released from the HLA complex on the affinity columns by addition of 10% acetic acid, and further separated from heavy chains and β2-microglobuline by boiling and passing the suspension through a 3-kDa cut-off membrane filter. Peptide pools from drug-treated and non-treated cells were separated by reverse phase high-pressure liquid chromatography (RP-HPLC) and fractions were collected. Lyophilized pre-fractionated samples were re-dissolved and analyzed by nano liquid chromatography-tandem mass spectrometry (LC-MS/MS) as described in the “B721-5701 protein lysate and LC-MS/MS analysis” section above.

**Identification of HLA-bound peptides using LC–MS/MS and analysis:**

Peptide identity was determined using mass tolerance of 10 ppm for precursor ion and of 0.05 Da for fragment ions subject to stringent bioinformatic parameters using PEAKS 8.5 or X software with a false discovery rate (FDR) cutoff of 5%. Post translational modifications (PTM) such as N-acetylation, methionine oxidation, cysteinylation and citrullination were considered. The filtered data set was further analyzed manually to exclude redundant peptides and recognized contaminants. Haptenated-peptides were identified in the HLA peptide datasets by using FLX PTM of (+453.06). LC-MS/MS spectra were also manually examined for FLX-related ions with z=1+ charge of +454.0689 (protonated FLX) or +160.0446 or +295.0382 representing FLX fragments). FLX modified peptides also contained MS2 fragments with +294 on lysines that supported covalent linkage at specific lysine residues.

The predicted binding affinity of peptides to HLA-B*57:01 was predicted using the NetMHCcons 1.0 Server (4). The frequency of each amino acid in the peptide sequence was assessed by Gibbs cluster 2.0 software (DTU, Denmark) (5). Gene Ontology pathways were evaluated using ‘Ingenuity Pathway Analysis (IPA) (6).

**Frequency of the proteome identified proteins in the HLA immunopeptidome and peptide sampling**:

The intensities of proteins for which peptides were identified in the immunopeptidome, from the MaxQuant output, were averaged and used as an estimation of abundance. Proteins were cross-referenced with their Uniprot accessions to extract their length (database downloaded May, 5, 2020) (7). Abundance data were log2-transformed and compared to the number of HLA peptides in the immunoproteome. Graphs were created in R (4.0.0) using ggplot2 (3.3.1) (8, 9). Linear trendlines for figures 3A and 3B, were fitted in quartile sections. ANCOVAs were run on each quartile using the zoo (1.8-8), reshape2 (1.4.4), and car (3.0-8) R packages (10-12).

HLA sampling density (D) was calculated according to a priory expectation as stated in Hoof et al. and Bassani-Sternberg et al. in order to account for non-unique peptide sequences (13) (14). To model the expected sampling density (D’) given the abundance and length of a protein, proteins were divided into 200 bins according to their abundance. We find the average D of each bin and fit a sigmoid function to this that can be used to calculate D’ for an individual protein. Fold over-presentation can then be represented as D/D’. Correlation coefficients were calculated in R.

**Synthesis of FLX-modified lysine residues**:

FLX-conjugated lysine residues were prepared as per Scornet et al’s method (1). Specialty and common chemical reagents and solvents including FLX sodium, 4-methoxybenzyl chloride, Fmoc-Lys-OH hydrochloride *N^α^*-Fmoc-L-lysine allyl ester hydrochloride (**3**), *N,N*-diisopropylethylamine (DIEA), tetrakis (triphenylphosphine)-palladium(0), triphenylphosphine, barbituric acid, anhydrous sodium sulfate, sodium bicarbonate, anhydrous *N,N*-dimethylformamide, anhydrous dichloromethane, diethyl ether, absolute ethanol, methanol, hexane, ethyl acetate and deuterated solvents (CDCl_3_, DMSO-*d*_6_) were all purchased from reputable commercial sources and used as received.

Flash chromatography purifications were performed on glass columns (2.5 cm or 6.0 cm I.D.) packed with silica gel 60 (230-400 mesh), whereas analytical thin-layer chromatography (TLC) analyses were conducted on 2.5 cm × 7.5 cm glass plates coated with a 0.25 mm thick layer of silica gel 60 F254.

All NMR experiments were performed using a spectrometer operating at 300.13 and 75.47 MHz for one-dimensional ^1^H and ^1^H-decoupled ^13^C, respectively. Samples were maintained at a temperature of 298 °K. All spectra were recorded in deuterated solvents and chemical shifts, δ, were reported in parts per million (ppm) relative to appropriate internal references. Elemental composition of new compounds is determined, under contract, by high resolution mass spectrometry (HRMS) using a Bruker Daltonics ApexQ FTICR mass spectrometer equipped with a 12 T magnet. Electrospray ionization in positive ion mode was used to generate [M+H]^+^ and [M+Na]^+^ ions out of test samples (0.01 mg dissolved in 1 mL of 10 mM ammonium acetate in MeCN:H_2_O (1:1 v/v)). Spectra were externally calibrated using 0.5 mg/mL solution of CsI in water, which yielded a series of peaks in the mass range used for analysis (200-2000 m/z). GC-MS experiments were carried out using PerkinElmer Autosystem XL gas chromatography system coupled to PerkinElmer Clarus 560S mass spectrometer.

**4-Methoxybenzyl (*2S*,*5R*,*6R*)-6-(3-(2-chloro-6-fluorophenyl)-5-methylisoxazole-4-carboxamido)-3,3-dimethyl-7-oxo-4-thia-1-azabicyclo [3.2.0] heptane-2-carboxylate** **(2).** To a solution of sodium flucloxaxillin **1** (4.75 g, 10.0 mmol) in anhydrous DMF (20 mL) was added 4-methoxybenzyl chloride (2 mL, 15 mmol); the solution was stirred at 60 °C over a period of 3.5 h and then poured into water (200 mL). The mixture was extracted with ethyl ether (3 × 100 mL). The combined organic extracts were collected and washed with a saturated solution of sodium bicarbonate (100 mL); the organic layer was dried over anhydrous sodium sulfate, filtered and rotoevaporated under reduced pressure to a sticky solid. The crude material was purified by silica gel chromatography using a gradient of EtOAc (0→50%) in hexane as the eluent. Fractions containing the pure product were collected and rotoevaporated under vacuum affording the pure flucloxacillin ester **2** (4.89 g, 8.5 mmol) as a white foam in a yield of 85%.^1^H NMR (300 MHz, DMSO-*d_6_*) δ: 8.93 (d, 1H, *J* = 7.2 Hz), 7.59 (m, 1H), 7.48 (m, 1H), 7.42 (m, 1H), 7.35 (d, 2H, *J* = 6.7 Hz), 6.94 (d, 2H, *J* = 6.7 Hz), 5.57 (dd, 1H, *J* = 4.0 and 7.2 Hz), 5.48 (d, 1H, *J* = 4.0 Hz), 5.12 (d, 2H, *J* = 3.2 Hz), 4.39 (s, 1H), 3.75 (s, 3H), 2.68 (s, 3H), 1.53 (s, 3H), 1.32 (s, 3H). ^13^C NMR (175 MHz, DMSO-*d_6_*) δ: 173.0, 171.6, 167.7, 162.2, 160.5, 159.8, 158.9, 155.0, 134.3, 133.2, 131.1, 131.0, 127.5, 126.2, 115.3, 114.3, 114.2, 113.4, 70.3, 67.8, 67.1, 64.6, 59.4, 55.5, 31.2, 26.8, 13.2. +HRMS-ESI: calcd for C_27_H_35_ClFN_3_O_6_SNa (M+Na)^+^ 596.1029, found 596.1030.

**4-Methoxybenzyl (2R,4S)-2-((5S,12R)-5-((allyloxy)carbonyl)-14-(3-(2-chloro-6-fluo-rophenyl)-5-methylisoxazol-4-yl)-1-(9H-fluoren-9-yl)-3,11,14-trioxo-2-oxa-4,10,13-triazatetradecan-12-yl)-5,5-dimethylthiazolidine-4-carboxylate** (**4**). To the flucloxacillin ester **2** and *N^2^*-Fmoc-L-lysine allyl ester hydrochloride (**3**, 1.87 g, 4.00 mmol), was added anhydrous dichloromethane (16 mL). The reaction mixture was brought to a reflux; *N*,*N*-diisopropylethylamine (766 µL, 4.40 mmol) was then added to the refluxing mixture, using an automatic syringe, at the rate of 0.8 mL/h. After a total reflux time of 6 h the reaction mixture was rotoevaporated under reduced pressure to a gummy material, which was purified by silica gel chromatography. A gradient of EtOAc (33%→50%) in hexane was used as the eluent. Fractions containing the pure product were collected and rotoevaporated under vacuum to provide the pure product **4** (2.25 g, 2.29 mmol) as a yellow solid in a yield of 57%.^1^H NMR (300 MHz, DMSO-*d*_6_) δ: 8.20 (d, 1H, *J* = 8.5 Hz), 8.00 (m, 1H), 7.89 (d, 2H, *J* = 7.4 Hz), 7.78 (d, 1H, *J* = 7.7 Hz), 7.71 (d, 2H, *J* = 7.4 Hz), 7.53 (m, 1H), 7.44-6.95 (m, 9H), 6.92 (d, 2H, *J* = 8.3 Hz), 5.93-5.82 (1H, m), 5.30 (d, 1H, *J* = 17.2 Hz), 5.20-5.12 (m, 2H), 4.99 (d, 1H, *J* = 11.8 Hz), 4.79 (t, 1H, *J* = 8.2 Hz), 4.57 (d, 2H, *J* = 5.0 Hz), 4.39 (t, 1H, *J* = 8.8 Hz), 4.32-4.19 (m, 3H), 4.08 (m, 1H), 3.74 (s, 3H), 3.52 (d, 1H, *J* = 12.9 Hz), 2.99 (m, 2H), 2.64 (s, 3H), 1.64 (m, 2H), 1.43 (s 3H), 1.32 (m, 4H), 1.02 (s, 3H). ^13^C NMR (175 MHz, DMSO-*d*_6_) δ: 172.6, 170.9, 170.8, 169.2, 169.1, 160.4, 159.7, 158.9, 156.6, 155.0, 144.3, 144.2, 141.1, 134.3, 132.8, 130.9, 130.8, 128.1, 127.9, 127.5, 126.0, 125.6, 120.5, 118.1, 117.1, 116.8, 115.2, 114.9, 114.2, 114.1, 72.1, 67.2, 66.5, 66.0, 65.2, 60.2, 59.2, 57.6, 55.5, 55.4, 54.3, 47.1, 38.6, 30.7, 28.7, 28.1, 27.4, 23.2, 21.2, 14.5, 12.9. +HRMS-ESI: calcd for C_51_H_53_ClFN_5_O_10_S (M+H)^+^ 982.3258, found 982.3267.

***N^2^*-(((9H-fluoren-9-yl)methoxy)carbonyl)-*N^6^*-((R)-2-(3-(2-chloro-6-fluorophenyl)-5-methylisoxazole-4-carboxamido)-2-((2R,4S)-4-(((4-methoxybenzyl)oxy)carbonyl)-5,5-dimethylthiazolidin-2-yl)acetyl)-L-lysine (5).** To a suspension of barbituric acid (307 mg, 2.40 mmol) in absolute ethanol (6 mL) was added a solution of the L-lysine conjugate **4** (1.96 g, 2.00 mmol) in anhydrous dichloromethane (12 mL) followed by catalytic amounts of tetrakis(triphenylphosphine)-palladium(0) (69 mg, 0.06 mmol) and triphenyphosphine (8 mg, 0.03 mmol). The reaction mixture was stirred under an inert atmosphere of argon over a period of 1 h at ~23 °C and then concentrated under reduced pressure to ~80% of its original volume. Insoluble material was filtered; the filtrate was rotoevaporated to dryness. The residue was purified by silica gel chromatography using a gradient of methanol (0%→2%) in dichloromethane as the eluent to provide the L-lysine-conjugate **5** (1.06 g, 1.13 mmol) as a yellow foam in a yield of 56%. ^1^H NMR (DMSO-*d*_6_) δ: 12.5 (s br, 1H), 8.18 (d, 1H, *J* = 8.7 Hz), 8.00 (t, 1H, *J* = 5.5 Hz), 7.89 (d, 2H, *J* = 7.4 Hz), 7.72 (d, 2H, *J* = 7.4 Hz), 7.65-7.50 (m, 3H), 7.44-7.28 (m, 8H), 6.92 (d, 2H, *J* = 8.7 Hz), 5.17 (d, 1H, *J* = 11.8 Hz), 5.00 (d, 1H, *J* = 11.8 Hz), 4.79 (t, 1H, *J* = 8.0 Hz), 4.40 (t, 1H, *J* = 8.8 Hz), 4.28-4.19 (m, 3H), 4.08 (m, 1H), 3.89 (m, 1H), 3.74 (s, 3H), 3.52 (d, 1H, *J* = 12.5 Hz), 2.99 (d br, 1H, *J* = 5.1 Hz), 2.65 (s, 3H), 1.67 (m, 1H), 1.58 (m, 1H), 1.43 (s, 3H), 1.33 (m, 4H), 1.23 (s, 3H). ^13^C NMR (DMSO-*d*_6_) δ: 174.5, 170.9, 162.2, 160.4, 159.7, 158.9, 156.6, 155.0, 144.3, 144.2, 141.1, 134.5, 134.4, 133.0, 132.8, 132.0, 130.9, 130.8, 128.1, 127.9, 127.5, 126.0, 125.7, 120.5, 117.1, 116.8, 115.2, 114.9, 114.2, 114.1, 72.1, 67.2, 66.5, 66.0, 59.2, 57.6, 55.5, 55.4, 55.3, 54.2, 47.1, 38.7, 30.8, 28.8, 28.1, 27.4, 23.3, 12.9. +HRMS-ESI: calcd for C_48_H_49_ClFN_5_O_10_S (M+H)^+^ 942.2945, found 942.2951.

The flucloxacillin-lysine conjugate is then used in the solid-phase peptide synthesis to provide the flucloxacillin peptide conjugate used in the proposed study.

**Peptide docking modeling**:

Coot (v0.8.9.2) (15) was used to replace the KAF11 peptide residues (PDB ID: 2YPK) with those of the RTKK peptide, LF9 peptide residues (PDB:2RFX) with those of KAA peptide and TW10 peptide residues (PDB:5V5M) with the TAA amino acids. The peptide structure was then docked with FlexPepDock (16, 17) using 100 low-and-high resolution structures. FLX (CID 21319) was fused to the resulting lowest energy peptide conformation at the corresponding lysine position using PyMOL (The PyMOL Molecular Graphics System v2.3.4, Schrödinger, LLC), and the resulting structures were energy minimized using OpenBabel (v3.0.0) (18) with the MMFF94 forcefield and steepest descent for a maximum of 2500 steps. Images were generated using PyMOL. For receptor modeling we used HIV KAF TcR (PDB ID: 2YPL) and KIR-3DL (PDB ID: 3VH8).

**Mice and *in vivo* treatment:**

HLA-B*57:01 Tg (19) and *H2-K^b^D^b^* knockout C57BL/6 (B6.129P2-*H2-Kb^tm1^H2-Db^tm1^* N12) (obtained from Taconic Biosciences, Hudson, NY) strains were backcrossed to generate that HLA-B*57:01 Tg / mouse MHC-I KO strain (Tg/KO) exclusively expressing HLA-B*57:01 as the MHC-I allele. Animals were phenotyped by flow cytometry as detailed below (also see Suppl Fig 2). Experimental mice were above 12 weeks of age. All mice were bred and housed under specific pathogen-free conditions in the animal facility of the NIAID, NIH (Rockville, MD) or in the AAALAC accredited animal facility of the U.S. FDA’s Division of Veterinary Medicine (Silver Spring, MD) in compliance with the Institutional Animal Care and Use Committee regulations of the U.S. FDA. Tg/KO mice were injected subcutaneously at the base of the tail on day 0 and day 7 with a mixture of 100 µg of FLX-haptenated peptide (FLX-peptide), 150 µg of HBV-co-peptide, a CD4+ helper T cell peptide, and IFA at a ratio of 1:1:2 (100 μL total volume). Control mice were treated with equal volume of Vehicle (WFI) lacking FLX-peptide. On day 14, mice were euthanized, and spleens were collected in sterile conditions for *in vitro* expansion.

***In vitro* cell cultures:**

*In vitro* T-cell responses to FLX-peptides were measured in cultures of total splenocytes of Tg/KO mice. Splenocyte suspensions were prepared by macerating the spleen through 70-μm cell strainers and lysis of red blood cells with Ammonium-Chloride-Potassium (ACK) lysing buffer (GIBCO). Cells were cultured for up to 5 days in complete RPMI (cRPMI) consisting of RPMI 1640 supplemented with 2 mM L-Glutamine, 100 U/mL Penicillin, and 100 μg/mL Streptomycin (all from GIBCO). During the first 2 days of culture, cRPMI was supplemented with 0.5% heat-inactivated, normal mouse serum (Equitech-Bio, Inc.) to reduce the background due to *in vivo* cell activation. At day 2, half of the media volume was replaced by fresh cRPMI containing 10% heat-inactivated, low-endotoxin FBS (Premium Select FBS, Atlanta Biologicals). Cells were seeded in 48-well tissue culture treated plates (Cat# 3548, Corning) at 3 x 10^6^ cells per well in 1 mL or in 96-well tissue culture treated plates (Cat# 3596, Corning) at 0.9 x 10^6^ cells per well in 0.3 mL and incubated at 37 °C and 5% CO_2_. Cells were left untreated or were cultured with 10 μg/mL of FLX-peptides or unmodified peptide (parent). Cultures without drug (None) served as a negative control. FLX-peptides were dissolved at 10 mg/mL in Water For Injection (WFI) for Cell Culture (GIBCO) at RT, aliquoted, and stored at -20°C.

At day 5 of the *in vitro* stimulation, secretion of IFN-γ was measured in cell culture supernatants by ELISA using commercial kits for mouse IFN-γ (eBiosciences). Production of IFN-γ and expression of PD-1 in T-lymphocytes were analyzed at the same time point by flow cytometry as detailed in the methods section below.

**Flow cytometry:**

Flow cytometry was used to phenotype the transgenic mice (Suppl Fig 2) and analyze immune cell subsets by intracellular and/or surface marker staining. Expression of the chimeric HLA molecule in T-lymphocytes, and B cells was measured by cell surface marker staining of blood. The blood was collected in MiniCollect 0.8 mL LH Lithium Heparin Sep. tubes (Cat# 450479, Greiner Bio-one GmbH) and transferred into 96-well sterile V-bottom plates (BRAND plates Cat# 781661, BRAND) for flow cytometry staining. Prior to staining, red blood cells were lysed with ACK lysing buffer as follows: one incubation with 75 μL of ACK /sample at RT for 5 min followed by a centrifugation (300 xg) at 4°C for 3 min and subsequently by a second incubation with 150 μL of ACK /sample at RT for 5 min. Neutralization of the ACK was accomplished by washing leukocytes twice in cold PBS (pH 7.4) without calcium chloride and magnesium chloride (GIBCO) supplemented with 5% heat-inactivated FBS. To prevent non-specific antibody (Ab) binding, cells were pre-incubated at 4°C for at least 15 min in 100 μL of Stain Buffer (BD Biosciences)/sample containing 10 μg/mL of anti-CD16/CD32 mAb (mouse Fc Block, clone 2.4G2, BD Biosciences). After blocking, samples were stained at 4°C for 30 min in the dark with 100 μL of a mixture constituted by 50% of Stain Buffer, 50% of Brilliant Stain Buffer (BD Horizon), and the appropriate diluted antibodies for cell surface markers. Once labeled, cells were washed by centrifugation at 4°C for 5 min with ACK Lysing buffer (150 μL per sample) and cold PBS supplemented with 5% heat-inactivated FBS (250 μL per sample) and finally resuspended in cold Stain Buffer prior to flow cytometry acquisition.

For intracellular detection of IFN-γ, cultures of 3 x 10^6^ splenocytes were left untreated or incubated with 10 μg/mL of FLX-peptide for approximately 5 days as detailed in the method section “*In vitro* culture assays”. During the last 8h of culture, cells were treated with Brefeldin A Solution (eBioscience) at a final dilution of 1/1000 and then tested. Viability was assessed by LIVE/DEAD Fixable Aqua Dead Cell Stain Kit (Invitrogen) following manufacturer’s directions while non-specific Ab binding was blocked with mouse Fc Block. After blocking, cells were stained for cell surface markers as indicated above, fixed, permeabilized using the BD Cytofix/Cytoperm Plus kit (BD Biosciences) as per manufacturer’s instructions, and finally stained at 4°C for 30 min in the dark with anti-mouse IFN-γ Ab. Once labeled, cells were washed twice with cold 1x BD Perm/Wash buffer (250 μl per sample) and finally resuspended in cold Stain Buffer prior to flow cytometry acquisition.

Antibodies used for staining were: anti-HLA B/C PE (clone B1.23.2, eBioscience), anti-H-2D[d] FITC (clone 34-2-12, BD Pharmingen), anti-CD3 APC/Cy7 (clone: 17A2, Biolegend), anti-CD4 BV605 (clone: RM4-5, BD Horizon), anti-CD8a BV711 (clone: 53-6.7, BD Horizon), anti-CD19 Alexa Fluor 700 (clone: eBio1D3 (1D3), eBioscience), anti-CD279 (PD-1) BV785 (clone: 29F.1A12, Biolegend), anti-IFN-γ APC (clone: XMG1.2, eBioscience). All antibodies were titered and tested alongside their specific iso3type controls and fluorescence minus one control samples in preliminary experiments.

Flow cytometry data acquisition and analysis were performed using LSR Fortessa X20-SORP and Diva 6.2/8.0.2 (BD Biosciences). Daily calibration and QC were performed using CS&T Beads (Cat# 642412 and 655051, BD Biosciences). CD4^+^ and CD8^+^ T-lymphocytes were pre-gated on live singlets CD3^+^ cells; B cells were pre-gated on live singlets CD3^-^ cells and identified as B220^+^CD19^+^.

REFERENCES:

1. Scornet N, Delarue-Cochin S, Azoury ME, Le Mignon M, Chemelle J-A, Nony E, et al. Bioinspired design and oriented synthesis of immunogenic site-specifically penicilloylated peptides. *Bioconjugate Chemistry* (2016) 27(11):2629-45. doi: 10.1021/acs.bioconjchem.6b00393

2. Chong C, Marino F, Pak H, Racle J, Daniel RT, Müller M, et al. High-throughput and sensitive immunopeptidomics platform reveals profound interferonγ-mediated remodeling of the Human Leukocyte Antigen (HLA) ligandome. *Mol Cell Proteomics* (2018) 17(3):533-48. doi: 10.1074/mcp.TIR117.000383

3. Norcross MA, Luo S, Lu L, Boyne MT, Gomarteli M, Rennels AD, et al. Abacavir induces loading of novel self-peptides into HLA-B*57: 01 an autoimmune model for HLA-associated drug hypersensitivity. *AIDS* (2012) 26(11):F21-F9. doi: 10.1097/QAD.0b013e328355fe8f

4. Andreatta M, Nielsen M. Gapped sequence alignment using artificial neural networks: application to the MHC class I system. *Bioinformatics* (2016) 32(4):511-7. doi: 10.1093/bioinformatics/btv639.

5. Andreatta M, Alvarez B, Nielsen M. GibbsCluster: unsupervised clustering and alignment of peptide sequences. *Nucleic Acids Research* (2017) 45(W1):W458-W63. doi: 10.1093/nar/gkx248

6. Felciano RM, Bavari S, Richards DR, Billaud JN, Warren T, Panchal R, et al. Predictive systems biology approach to broad-spectrum, host-directed drug target discovery in infectious diseases. *Pac Symp Biocomput* (2013):17-28. PMID: 23424108

7. Consortium TU. UniProt: a worldwide hub of protein knowledge. *Nucleic Acids Research* (2018) 47(D1):D506-D15. doi: 10.1093/nar/gky1049

8. Wickham H. ggplot2: Elegant graphics for data analysis. *Springer-Verlag, New York* (2016)

9. R: A language and environment for statistical computing. R Foundation for Statistical Computing [Internet]. (2013). Available from: <http://www.R-project.org/>

10. Zeileis A, Grothendieck G. zoo: S3 infrastructure for regular and irregular time series. *Journal of Statistical Software* (2005) 14(6):27. doi: 10.18637/jss.v014.i06

11. Wickham H. Reshaping Data with the reshape Package. *Journal of Statistical Software* (2007) 21(12):20. doi: 10.18637/jss.v021.i12

12. Fox JaW, Sanford. An {R} companion to applied regression. *Third Edition Thousand Oaks CA: Sage* (2019)

13. Bassani-Sternberg M, Pletscher-Frankild S, Jensen LJ, Mann M. Mass spectrometry of Human Leukocyte Antigen Class I peptidomes reveals strong effects of protein abundance and turnover on antigen presentation. *Molecular & Cellular Proteomics* (2015) 14(3):658-73. doi: 10.1074/mcp.M114.042812

14. Hoof I, van Baarle D, Hildebrand WH, Keşmir C. Proteome sampling by the HLA Class I Antigen processing pathway. *PLoS Comput Biol* (2012) 8(5):e1002517. doi: 10.1371/journal.pcbi.1002517

15. Emsley P, Lohkamp B, Scott WG, Cowtan K. Features and development of Coot. *Acta Crystallogr* (2010) 66:486-501. doi: 10.1107/s0907444910007493

16. London N, Raveh B, Cohen E, Fathi G, Schueler-Furman O. Rosetta FlexPepDock web server-high resolution modeling of peptide-protein interactions. *Nucleic Acids Res* (2011):W249-53.. doi: 10.1093/nar/gkr431

17. Raveh B, London N, Schueler-Furman O. Sub-angstrom modeling of complexes between flexible peptides and globular proteins. *Proteins* (2010) 78(9):2029-40. Epub 2010/05/11. doi: 10.1002/prot.22716

18. O'Boyle NM, Banck M, James CA, Morley C, Vandermeersch T, Hutchison GR. Open Babel: An open chemical toolbox. *J Cheminform* (2011) 3:33. doi: 10.1186/1758-2946-3-33

19. Cardone M, Garcia K, Tilahun ME, Boyd LF, Gebreyohannes S, Yano M, et al. A transgenic mouse model for HLA-B*57:01–linked abacavir drug tolerance and reactivity. *The Journal of Clinical Investigation* (2018) 128(7):2819-32. doi: 10.1172/JCI99321
