# Supplementary material for: Alterations in the HLA-B*57:01 Immunopeptidome by Flucloxacillin and Immunogenicity of Drug-Haptenated Peptides
Source: Front Immunol. 2021 Feb 9;11:629399. doi: 10.3389/fimmu.2020.629399 (PMC7900192; doi:10.3389/fimmu.2020.629399)
Supplement: Supplementary file 2 [file Presentation_1.pptx]

## Slide 1
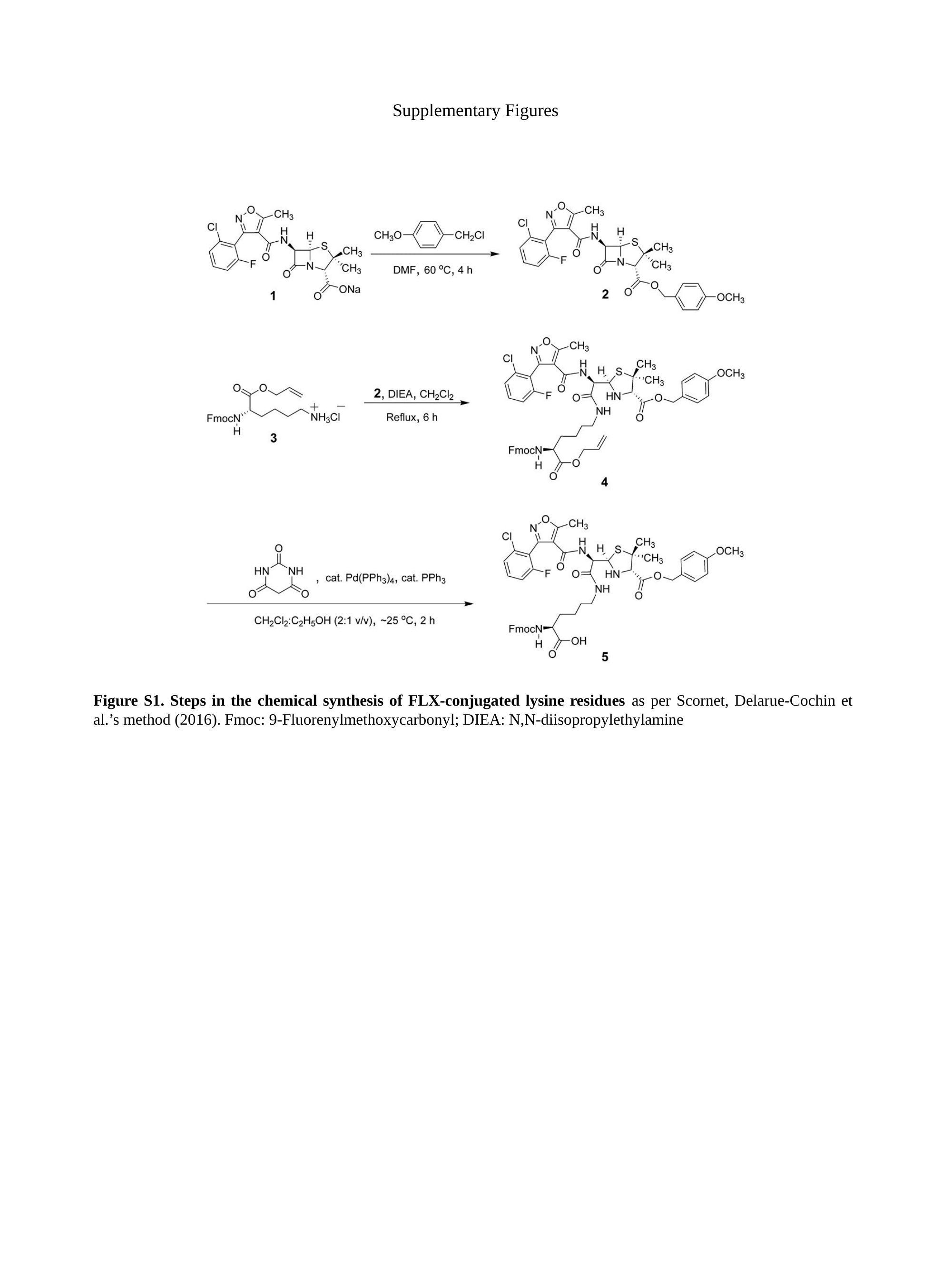

Supplementary Figures
Figure S1. Steps in the chemical synthesis of FLX-conjugated lysine residues as per Scornet, Delarue-Cochin et al.’s method (2016). Fmoc: 9-Fluorenylmethoxycarbonyl; DIEA: N,N-diisopropylethylamine

## Slide 2
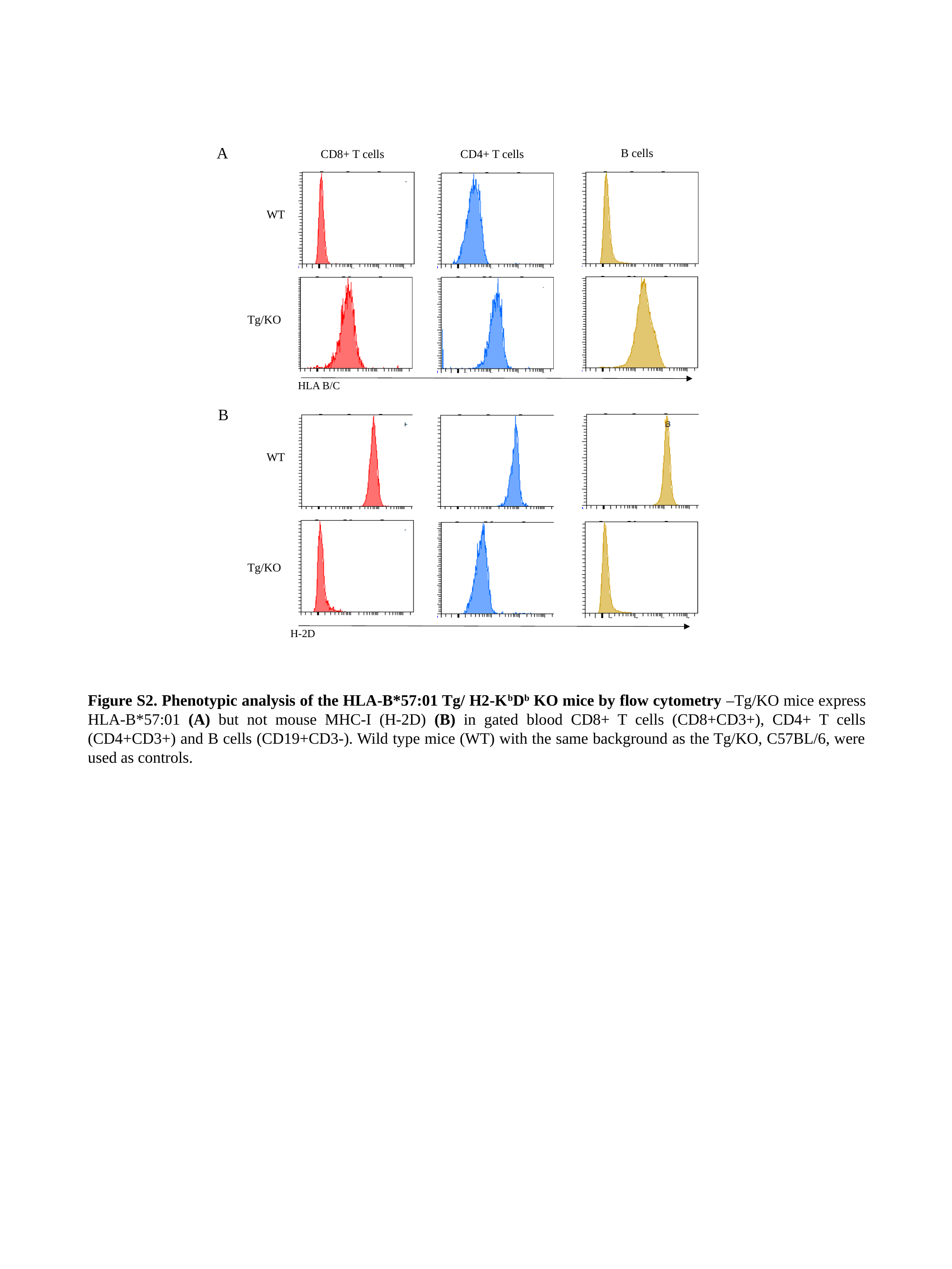

A
B cells
CD8+ T cells
CD4+ T cells
WT
Tg/KO
HLA B/C
B
B
/
WT
Tg/KO
H-2D
Figure S2. Phenotypic analysis of the HLA-B*57:01 Tg/ H2-KbDb KO mice by flow cytometry –Tg/KO mice express HLA-B*57:01 (A) but not mouse MHC-I (H-2D) (B) in gated blood CD8+ T cells (CD8+CD3+), CD4+ T cells (CD4+CD3+) and B cells (CD19+CD3-). Wild type mice (WT) with the same background as the Tg/KO, C57BL/6, were used as controls.

## Slide 3
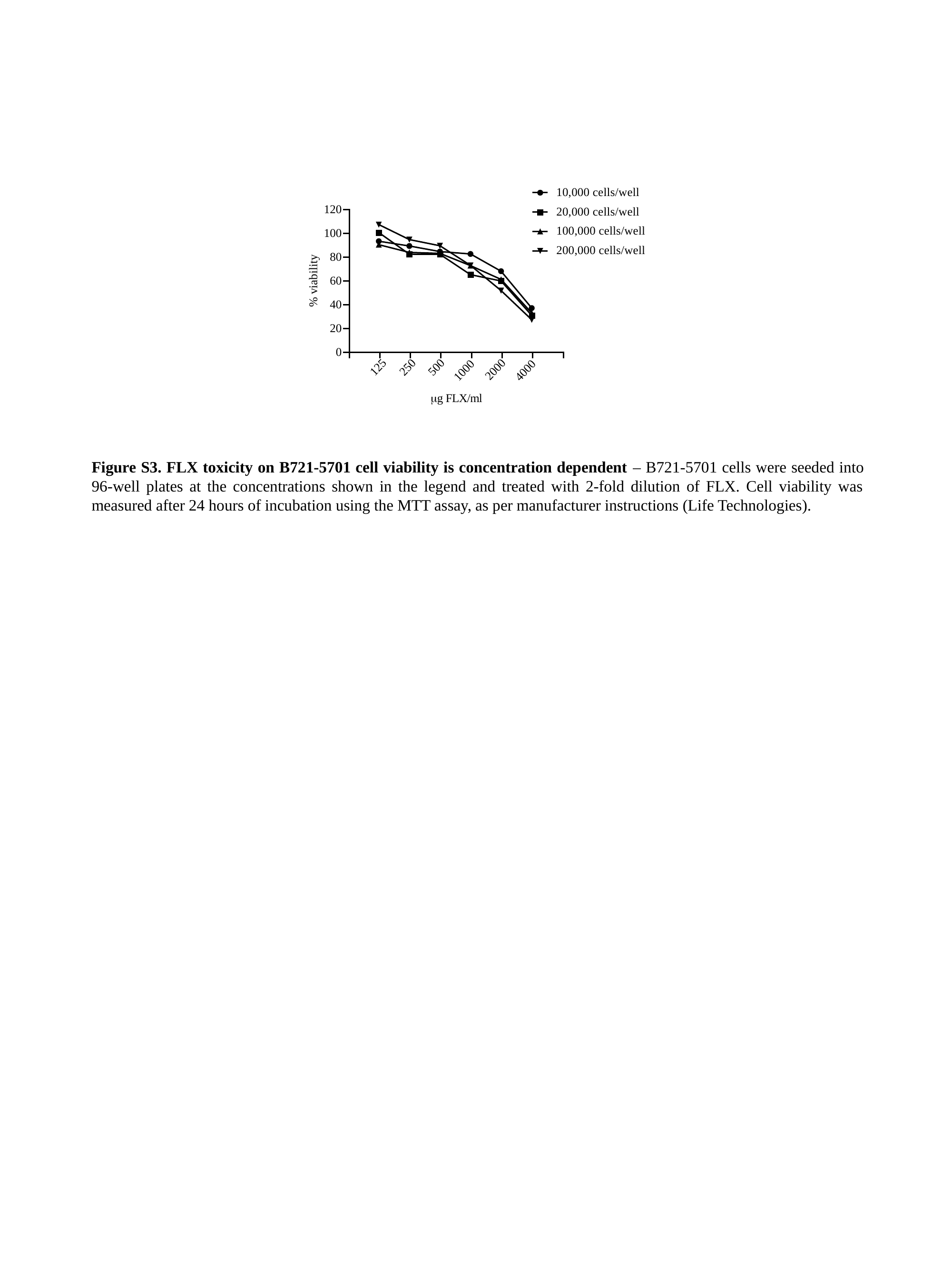

4000
125
250
500
1000
2000
Figure S3. FLX toxicity on B721-5701 cell viability is concentration dependent – B721-5701 cells were seeded into 96-well plates at the concentrations shown in the legend and treated with 2-fold dilution of FLX. Cell viability was measured after 24 hours of incubation using the MTT assay, as per manufacturer instructions (Life Technologies).

## Slide 4
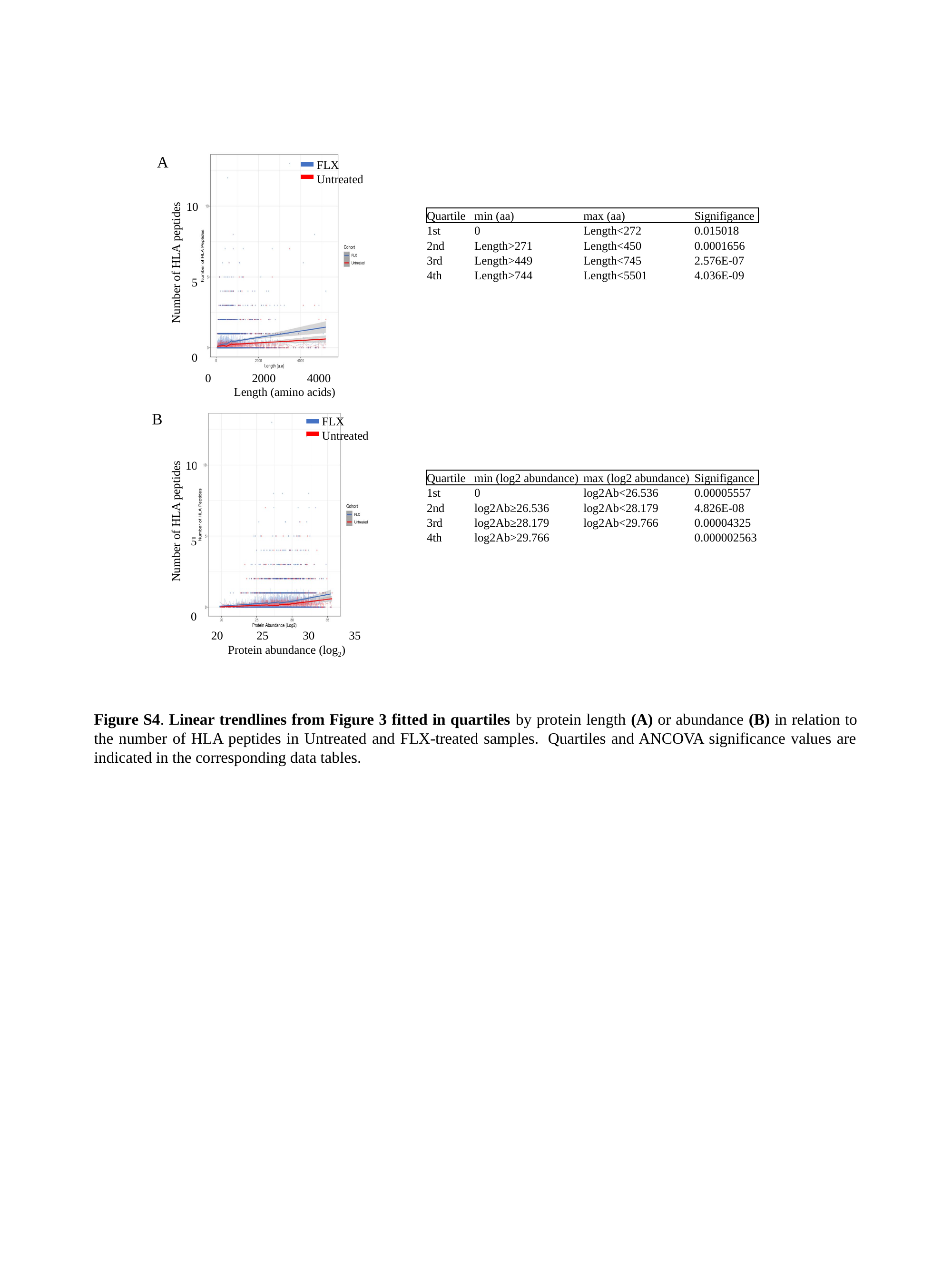

FLX
 Untreated
10
Number of HLA peptides
5
0
0
2000
4000
Length (amino acids)
A
| Quartile | min (aa) | max (aa) | Signifigance |
| --- | --- | --- | --- |
| 1st | 0 | Length<272 | 0.015018 |
| 2nd | Length>271 | Length<450 | 0.0001656 |
| 3rd | Length>449 | Length<745 | 2.576E-07 |
| 4th | Length>744 | Length<5501 | 4.036E-09 |
 FLX
 Untreated
10
Number of HLA peptides
5
0
20
25
30
35
Protein abundance (log2)
B
| Quartile | min (log2 abundance) | max (log2 abundance) | Signifigance |
| --- | --- | --- | --- |
| 1st | 0 | log2Ab<26.536 | 0.00005557 |
| 2nd | log2Ab≥26.536 | log2Ab<28.179 | 4.826E-08 |
| 3rd | log2Ab≥28.179 | log2Ab<29.766 | 0.00004325 |
| 4th | log2Ab>29.766 | | 0.000002563 |
Figure S4. Linear trendlines from Figure 3 fitted in quartiles by protein length (A) or abundance (B) in relation to the number of HLA peptides in Untreated and FLX-treated samples.  Quartiles and ANCOVA significance values are indicated in the corresponding data tables.

## Slide 5
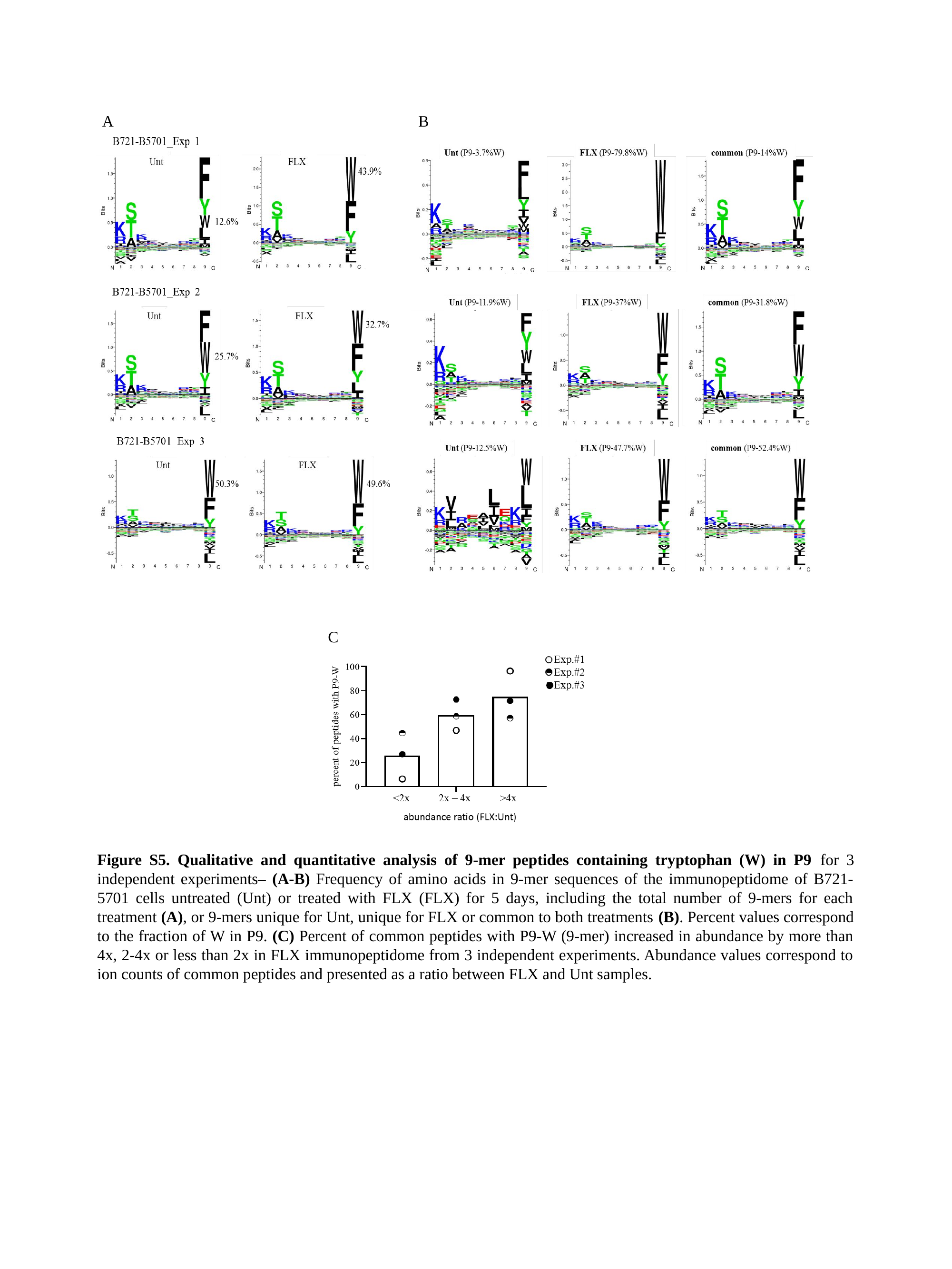

A
B
C
Figure S5. Qualitative and quantitative analysis of 9-mer peptides containing tryptophan (W) in P9 for 3 independent experiments– (A-B) Frequency of amino acids in 9-mer sequences of the immunopeptidome of B721-5701 cells untreated (Unt) or treated with FLX (FLX) for 5 days, including the total number of 9-mers for each treatment (A), or 9-mers unique for Unt, unique for FLX or common to both treatments (B). Percent values correspond to the fraction of W in P9. (C) Percent of common peptides with P9-W (9-mer) increased in abundance by more than 4x, 2-4x or less than 2x in FLX immunopeptidome from 3 independent experiments. Abundance values correspond to ion counts of common peptides and presented as a ratio between FLX and Unt samples.

## Slide 6
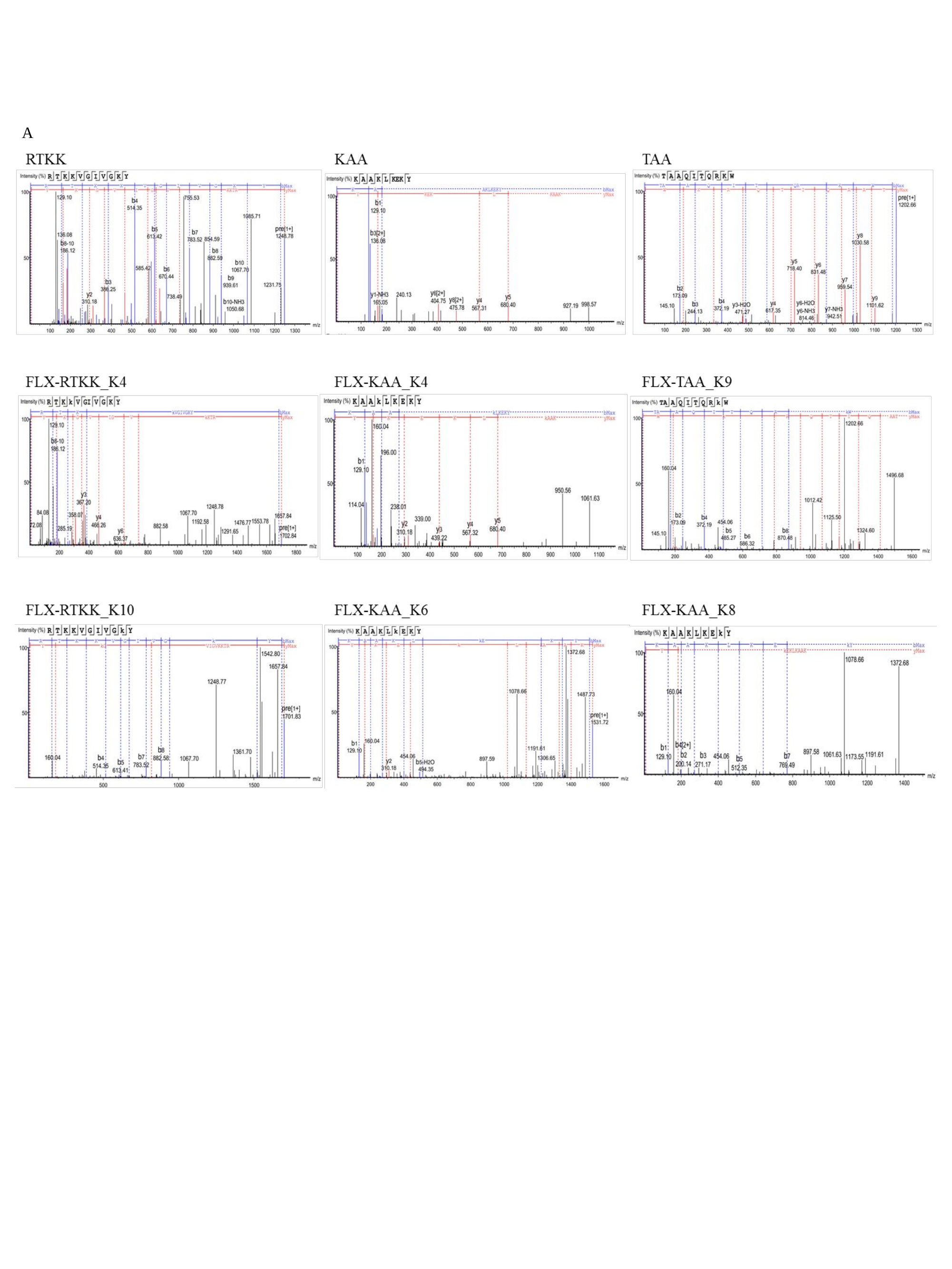

A

## Slide 7
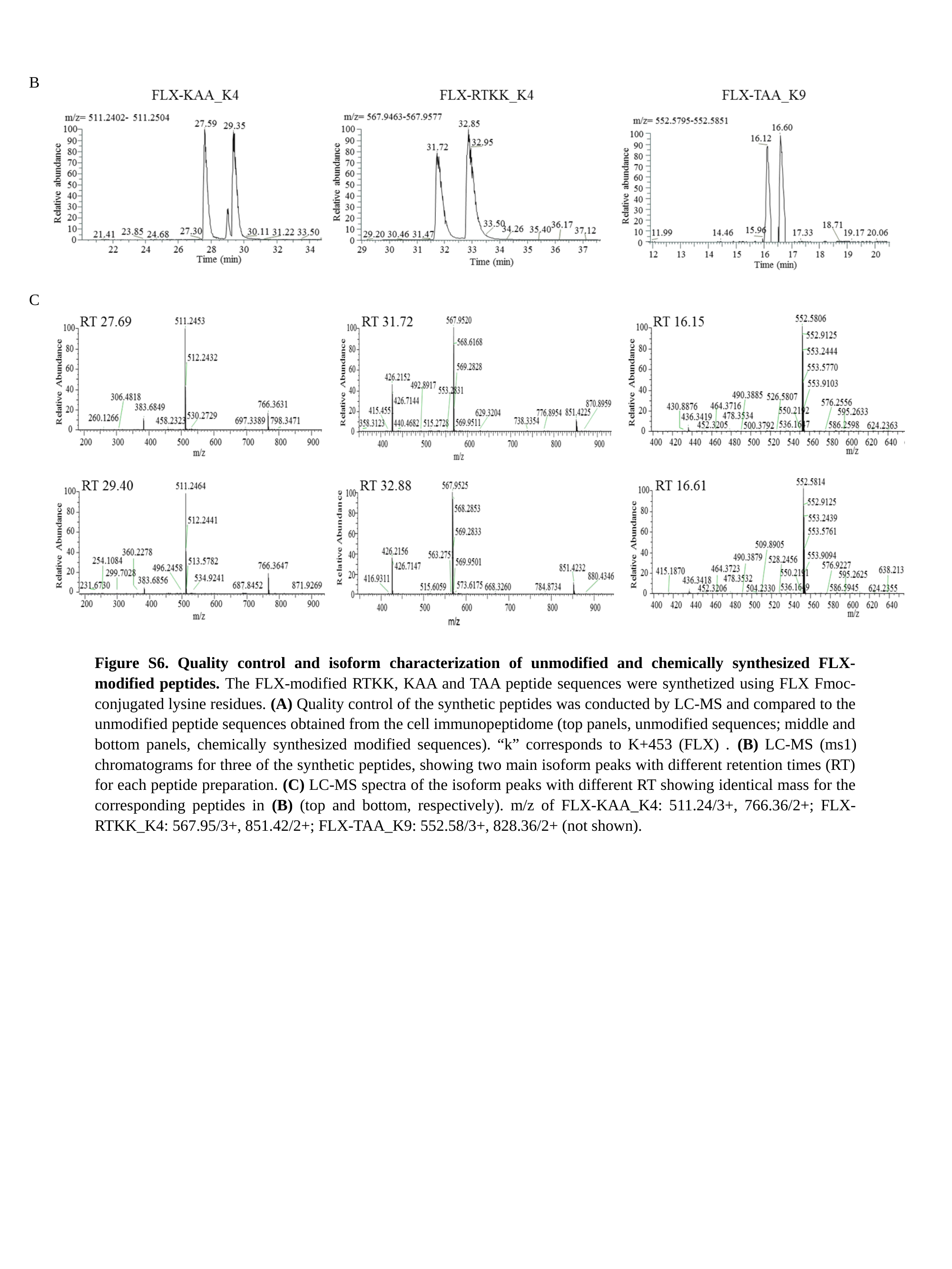

B
C
Figure S6. Quality control and isoform characterization of unmodified and chemically synthesized FLX-modified peptides. The FLX-modified RTKK, KAA and TAA peptide sequences were synthetized using FLX Fmoc-conjugated lysine residues. (A) Quality control of the synthetic peptides was conducted by LC-MS and compared to the unmodified peptide sequences obtained from the cell immunopeptidome (top panels, unmodified sequences; middle and bottom panels, chemically synthesized modified sequences). “k” corresponds to K+453 (FLX) . (B) LC-MS (ms1) chromatograms for three of the synthetic peptides, showing two main isoform peaks with different retention times (RT) for each peptide preparation. (C) LC-MS spectra of the isoform peaks with different RT showing identical mass for the corresponding peptides in (B) (top and bottom, respectively). m/z of FLX-KAA_K4: 511.24/3+, 766.36/2+; FLX-RTKK_K4: 567.95/3+, 851.42/2+; FLX-TAA_K9: 552.58/3+, 828.36/2+ (not shown).

## Slide 8
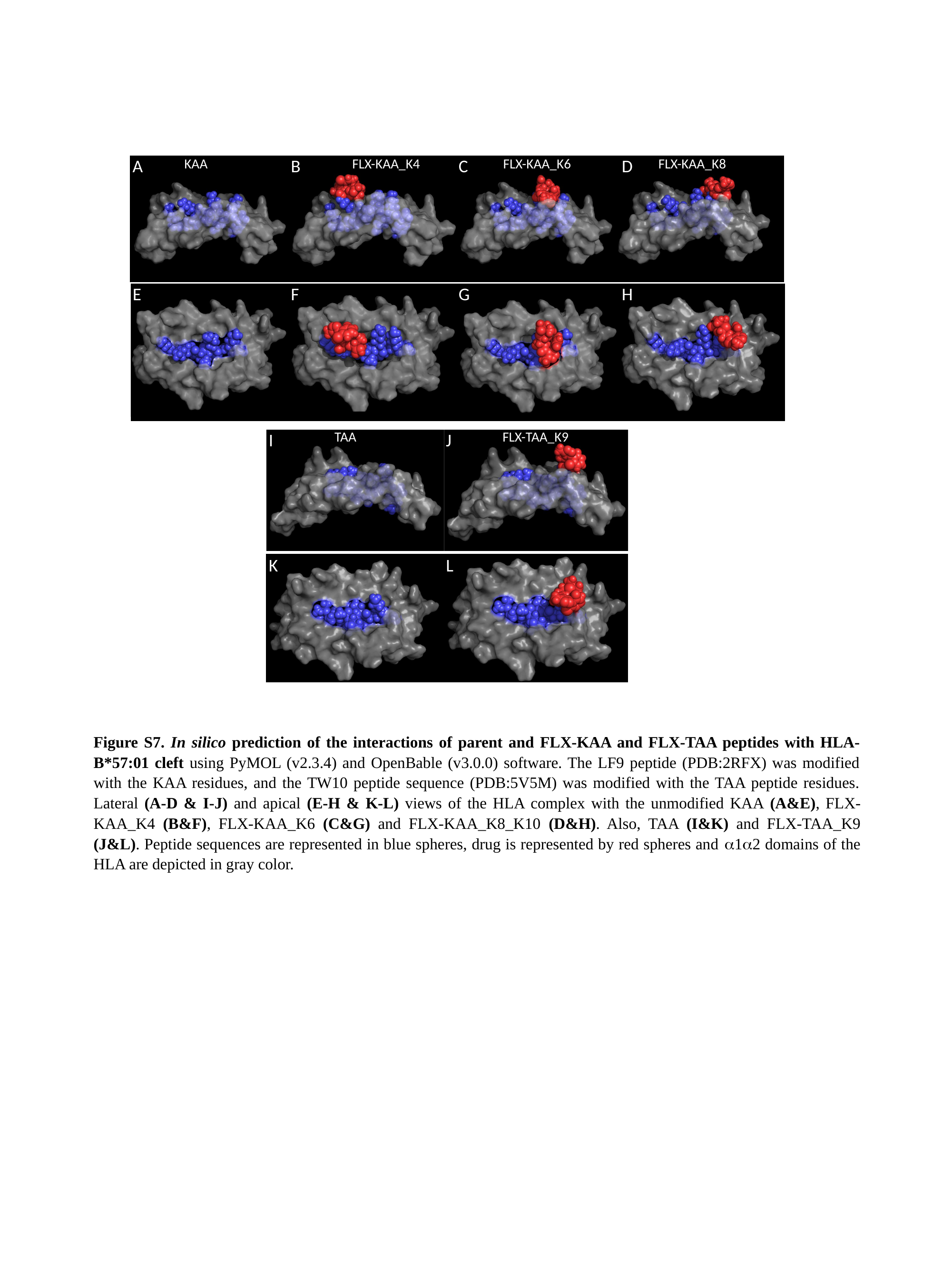

A
B
C
D
KAA
FLX-KAA_K4
FLX-KAA_K6
FLX-KAA_K8
E
F
G
H
TAA
FLX-TAA_K9
I
J
K
L
Figure S7. In silico prediction of the interactions of parent and FLX-KAA and FLX-TAA peptides with HLA-B*57:01 cleft using PyMOL (v2.3.4) and OpenBable (v3.0.0) software. The LF9 peptide (PDB:2RFX) was modified with the KAA residues, and the TW10 peptide sequence (PDB:5V5M) was modified with the TAA peptide residues. Lateral (A-D & I-J) and apical (E-H & K-L) views of the HLA complex with the unmodified KAA (A&E), FLX-KAA_K4 (B&F), FLX-KAA_K6 (C&G) and FLX-KAA_K8_K10 (D&H). Also, TAA (I&K) and FLX-TAA_K9 (J&L). Peptide sequences are represented in blue spheres, drug is represented by red spheres and α1α2 domains of the HLA are depicted in gray color.
