# Supplementary material for: Alterations in the HLA-B*57:01 Immunopeptidome by Flucloxacillin and Immunogenicity of Drug-Haptenated Peptides
Source: Front Immunol. 2021 Feb 9;11:629399. doi: 10.3389/fimmu.2020.629399 (PMC7900192; doi:10.3389/fimmu.2020.629399)
Supplement: Supplementary file 3 [file Presentation_2.pptx]

## Slide 1
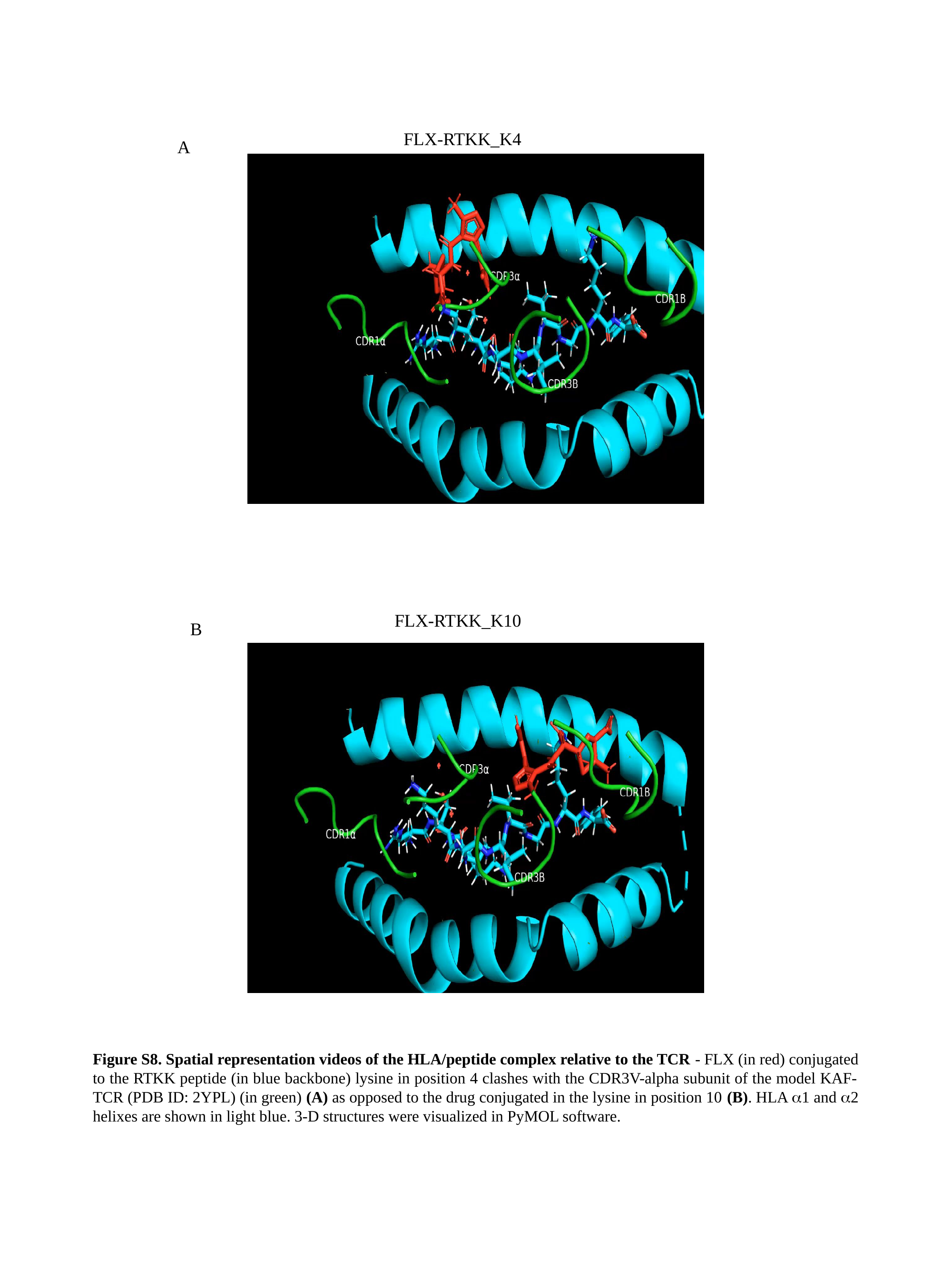

FLX-RTKK_K4
A
FLX-RTKK_K10
B
Figure S8. Spatial representation videos of the HLA/peptide complex relative to the TCR - FLX (in red) conjugated to the RTKK peptide (in blue backbone) lysine in position 4 clashes with the CDR3V-alpha subunit of the model KAF-TCR (PDB ID: 2YPL) (in green) (A) as opposed to the drug conjugated in the lysine in position 10 (B). HLA α1 and α2 helixes are shown in light blue. 3-D structures were visualized in PyMOL software.
