# Supplementary material for: Alterations in the HLA-B*57:01 Immunopeptidome by Flucloxacillin and Immunogenicity of Drug-Haptenated Peptides
Source: Front Immunol. 2021 Feb 9;11:629399. doi: 10.3389/fimmu.2020.629399 (PMC7900192; doi:10.3389/fimmu.2020.629399)
Supplement: Supplementary file 4 [file Presentation_3.pptx]

## Slide 1
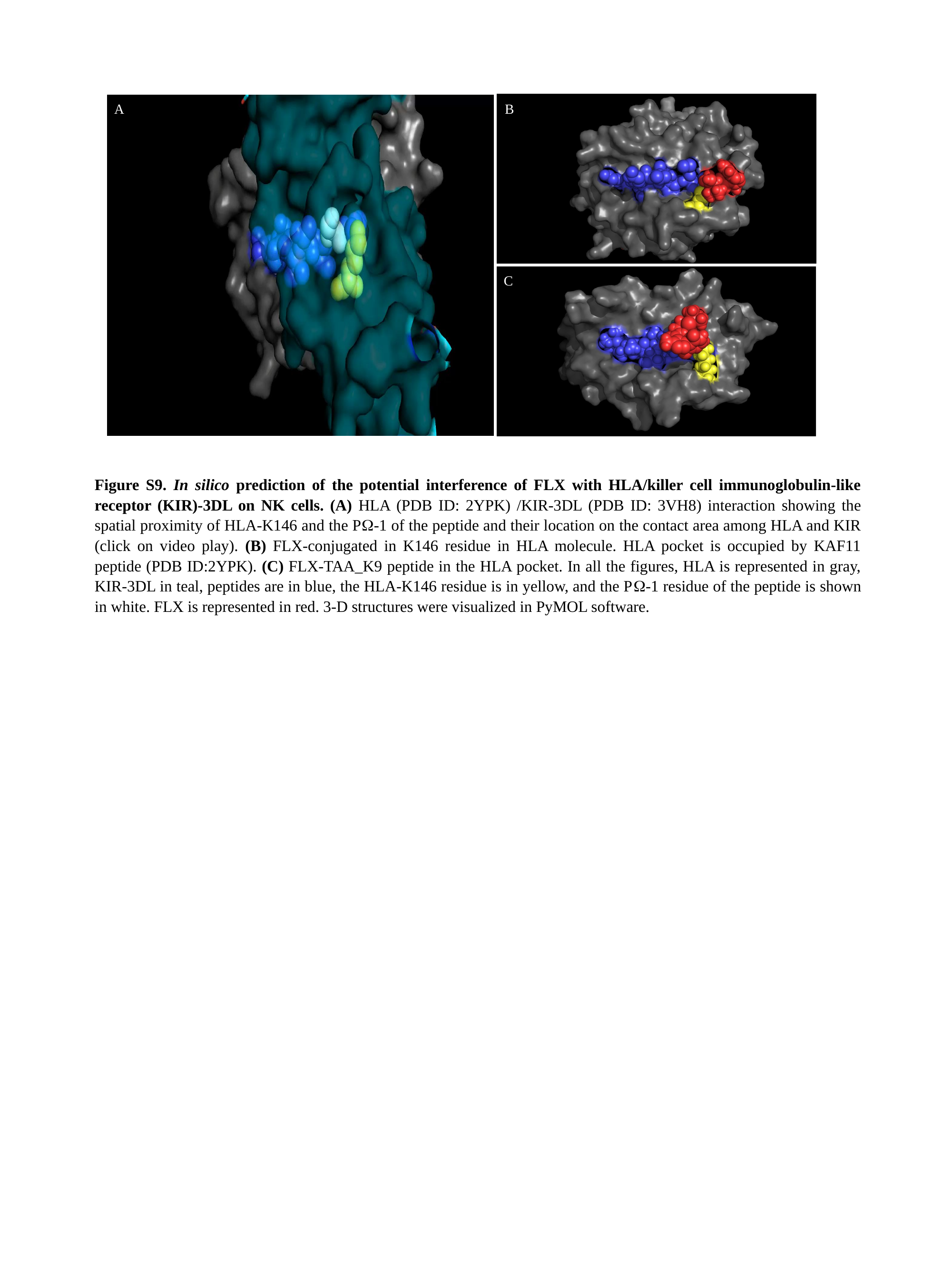

A
B
C
Figure S9. In silico prediction of the potential interference of FLX with HLA/killer cell immunoglobulin-like receptor (KIR)-3DL on NK cells. (A) HLA (PDB ID: 2YPK) /KIR-3DL (PDB ID: 3VH8) interaction showing the spatial proximity of HLA-K146 and the PΩ-1 of the peptide and their location on the contact area among HLA and KIR (click on video play). (B) FLX-conjugated in K146 residue in HLA molecule. HLA pocket is occupied by KAF11 peptide (PDB ID:2YPK). (C) FLX-TAA_K9 peptide in the HLA pocket. In all the figures, HLA is represented in gray, KIR-3DL in teal, peptides are in blue, the HLA-K146 residue is in yellow, and the PΩ-1 residue of the peptide is shown in white. FLX is represented in red. 3-D structures were visualized in PyMOL software.
